# Macrophage-derived exosomal aminopeptidase N aggravates sepsis-induced acute lung injury by regulating necroptosis of lung epithelial cell

**DOI:** 10.1038/s42003-022-03481-y

**Published:** 2022-06-06

**Authors:** Ting Gong, Xuedi Zhang, Zhiyong Peng, Yinfeng Ye, Ruimeng Liu, Yinggui Yang, Zhugui Chen, Zhihao Zhang, Hongfei Hu, Shuang Yin, Yi Xu, Jing Tang, Youtan Liu

**Affiliations:** 1 Department of Anesthesiology, Shenzhen Hospital, Southern Medical University, No. 1333, Xinhu Road, Baoan District, Shenzhen, 518110 Guangdong China; 2 Department of Anaesthetics, Affiliated Hospital of Guangdong Medical University, No. 57 People Avenue South, Zhanjiang, 524001 Guangdong China; 3 Department of Anesthesiology, Central People’s Hospital of Zhanjiang, No. 236 Yuanzhu Road, Zhanjiang, 524045 Guangdong China; 4 Department of Anesthesiology, The First People’s Hospital of Foshan, No. 81, Lingnan Avenue, Chancheng District, 528000 Foshan, China

**Keywords:** Sepsis, Necroptosis

## Abstract

Sepsis-induced acute lung injury (ALI) is a serious sepsis complication and the prevailing cause of death. Circulating plasma exosomes might exert a key role in regulating intercellular communication between immunological and structural cells, as well as contributing to sepsis-related organ damage. However, the molecular mechanisms by which exosome-mediated intercellular signaling exacerbate ALI in septic infection remains undefined. Therefore, we investigated the effect of macrophage-derived exosomal APN/CD13 on the induction of epithelial cell necrosis. Exosomal APN/CD13 levels in the plasma of septic mice and patients with septic ALI were found to be higher. Furthermore, increased plasma exosomal APN/CD13 levels were associated with the severity of ALI and fatality in sepsis patients. We found remarkably high expression of APN/CD13 in exosomes secreted by LPS-stimulated macrophages. Moreover, c-Myc directly induced APN/CD13 expression and was packed into exosomes. Finally, exosomal APN/CD13 from macrophages regulated necroptosis of lung epithelial cells by binding to the cell surface receptor TLR4 to induce ROS generation, mitochondrial dysfunction and NF-κB activation. These results demonstrate that macrophage-secreted exosomal APN/CD13 can trigger epithelial cell necroptosis in an APN/CD13-dependent manner, which provides insight into the mechanism of epithelial cell functional disorder in sepsis-induced ALI.

## Abstract (web summary)

Necroptosis of lung epithelial cells is regulated by aminopeptidase N levels in circulating plasma exosomes in patients and mice with sepsis-induced acute lung injury.

## Introduction

Sepsis is a potentially fatal organ failure syndrome induced by a dysregulated host response to infection, which results in acute organ dysfunction^1^. Sepsis-related deaths account for 11 million of the estimated 49 million patients who develop sepsis each year, accounting for 19.7% of all global deaths^2^, thus sepsis has become the main cause of deaths in intensive care unit (ICU). The lungs are considered as the most vulnerable target organs in sepsis to excessive inflammatory responses and are susceptible to acute lung injury (ALI) early in the course of the disease, making it a critical death cause in sepsis patients and a major threat to human health^3^. Therefore, understanding the pathophysiology of ALI is crucial for the comprehensive treatment of sepsis.

Exosomes secreted by various cell types naturally exist in body fluids without being degraded by enzymes, and mediate the progression of many diseases by mediating cell-to-cell signaling^4,5^. Under pathological conditions, donor cells load specific active substances, such as RNAs or proteins, into exosomes, which then translocate these active substances to recipient cells, leading to phenotypic variations in the recipient cells^6,7^. Exosomes also involve in the occurrence and advance of sepsis. Previous studies have confirmed that exosomes released from various effector cells are related to diverse tissue or organ damage in sepsis^8^. Gao et al. identified the characteristics of plasma exosomal miRNA profiles and revealed that circulating exosomes deliver miR-1-3p to endothelial cells, leading to the progression of sepsis-induced lung injury^9^. Furthermore, ALI mice have an abundance of serum exosomes in their peripheral blood, and these exosomes can transfer miR-155 into lung macrophages and activate them, resulting in lung injury^10^. Therefore, exosomes may act as potential clinical indicators of damaged tissues and organs during sepsis.

Exosomal contents, on the other hand, can remain stable in circulating plasma, making them attractive biomarkers for a variety of therapeutic applications. However, when compared to other diseases such as cancer, cardiovascular disease^11^ and ischemic stroke^12^, but only a few reports available on the exosomal systemic proteomic analysis, mainly plasma exosomes in patients with sepsis-induced ALI. In the present study, isolation of exosomes from the plasma of septic patients with ALI was performed by the process of ultracentrifugation. We then performed quantitative proteomic analyses to investigate the protein profile in the exosomes and revealed that exosomal APN/CD13 increased in the plasma of sepsis ALI patients; we confirmed that a similar increase occurs in septic mouse models.

Aminopeptidase N (APN/CD13) is a membrane-bound, Zn^2+^-dependent ectopeptidase that catalyzes the cleavage of short peptides’ N-terminal amino acids. APN/CD13 is regarded as a cancer-specific biomarker that is highly expressed on the cell membrane surface and mediates progression, invasion, and migration in various tumors, including acute myeloid leukemia, malignant pleural mesothelioma, and liver cancer^13–15^. Moreover, APN/CD13 does not only affect the development and differentiation of myeloid cells but also participates in the immune response and inflammation^16,17^. In addition, it has been shown that APN/CD13 inhibitors have clear anti-inflammatory effects^18,19^. However, few studies have explored whether plasma exosomal APN/CD13 participates in sepsis-induced ALI by mediating the communication of one cell to another.

We discovered that APN/CD13 transported by exosomes for the period of sepsis might be picked up by lung epithelial cells and trigger necroptosis in our investigation. Furthermore, circulating exosomal APN was mobilized to the lung tissue, which caused a significant inflammatory response and lung tissue injury in vivo. Moreover, patients with higher levels of plasma exosomal APN/CD13 exhibited a poor prognosis in sepsis. Interestingly, APN/CD13 is a multifunctional cell surface ectopeptidase that is highly expressed on monocytes and macrophages^20^. Previous research has suggested that CD13 expression levels in human peripheral blood monocytes increase in response to injury or treatment with lipopolysaccharides (LPS)^21^. Further studies have conclusively shown that APN/CD13 leads to increased reactive oxygen species (ROS) production by binding to TLR4^22^. In addition, CD13-mediated phagocytosis induces the production of ROS^23^. However, the molecular mechanisms revealing the effects of exosomal APN/CD13 in the development of lung epithelial cell necrosis and lung inflammation remain unclear.

Therefore, the present study was designed to investigate whether elevated exosomal APN/CD13 mediates the transmission of inflammatory information between macrophages and lung epithelial cells, exacerbating the development of ALI. We suggest that exosomal APN/CD13 may play a key role in inducing lung epithelial cell injury and may be a possible target for ALI in sepsis, which has important research significance and clinical application value.

## Results

### APN/CD13 is enriched in exosomes isolated from the plasma of sepsis-induced ALI patients

**Fig. 1:**
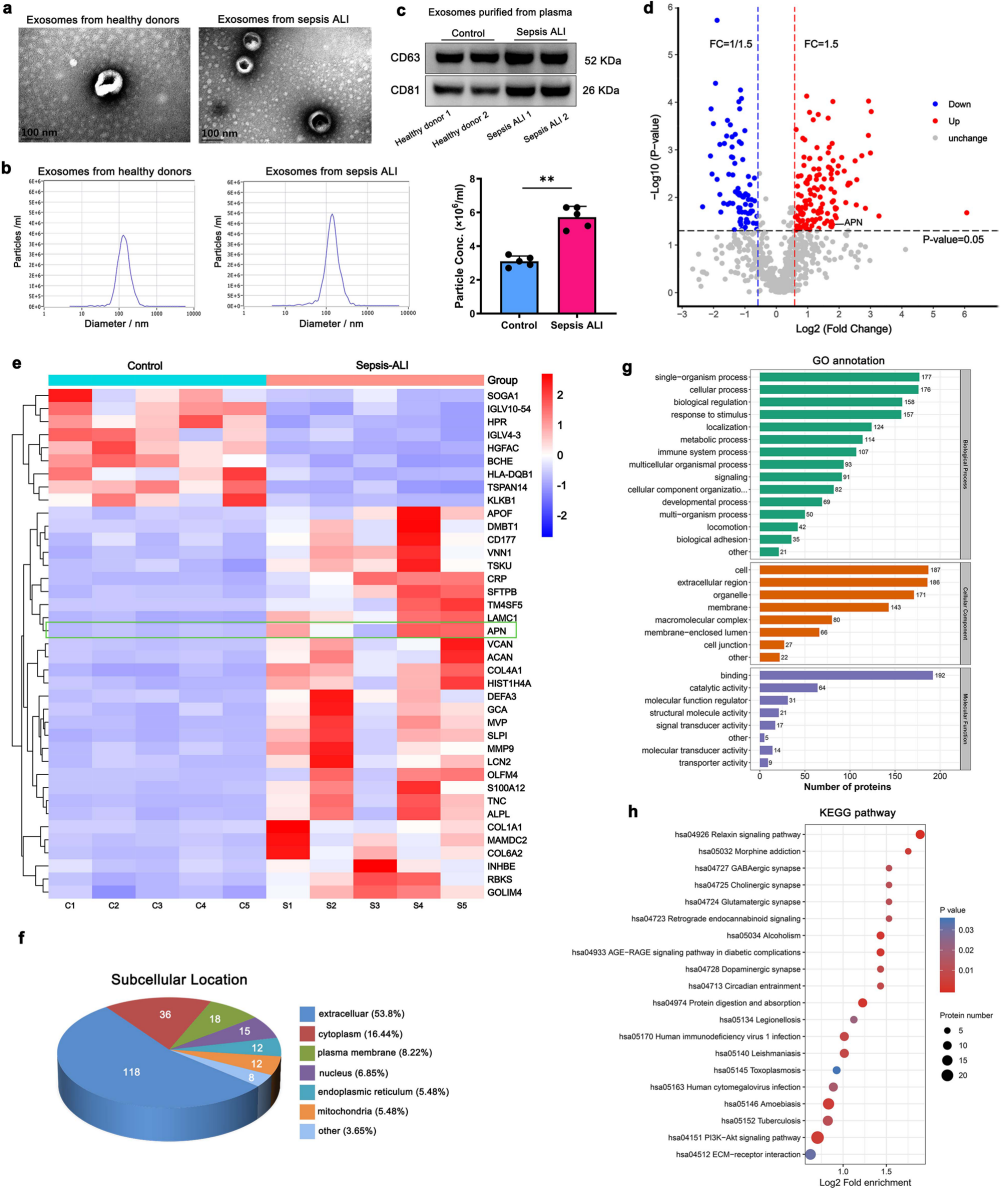
Proteomics analysis of plasma exosomes from the controls and sepsis-induced ALI patients. **a** Scanning electron microscopy images of plasma exosomes from healthy donors and patients with ALI caused by sepsis (scale bar: 100 nm). **b** Concentration (particles/mL) and modal size (nm) of exosome samples according to NTA measures. Mean ± SD (*n* = 5), **P < 0.01. **c** Exosome markers, including CD63 and CD81, were analyzed using western blot analysis. **d** Volcano plot showing differentially expressed protein in plasma exosomes from the healthy donors compared to sepsis-induced ALI patients. **e** Heatmap display showing 39 differentially expressed proteins from each group of the independent samples. **f** Differentially expressed proteins’ subcellular locations. **g** Gene ontology (GO) annotation was performed to characterize the biological functions of these differentially expressed proteins. **h** KEGG pathway enrichment analysis in cases vs. control group.

We have selected concentrations of plasma exosome in patients with ALI induced by sepsis (*n* = 5) and healthy donors (*n* = 5) from cohort 1 (Supplementary Table 1). Enriched preparations of exosomes from healthy donors were obtained using ultracentrifugation. To confirm the presence of isolated exosomes, we analyzed the size, morphology, and protein enrichment of the exosomes. Transmission electron microscopy (TEM) showed typical exosome morphology and cup-shaped vesicles with sizes 50–150 nm (Fig. 1a). The total number of exosomes was determined using NanoSight analysis (NTA), which revealed the higher plasma concentration of exosomes in the sepsis ALI group than in the control group (Fig. 1b). The frequently reported exosomal tetraspanine-markers CD63 and CD81 in either biological replicate for both patient groups were also confirmed by Western blot analysis of the harvested exosomes (Fig. 1c). Altogether, these results highlight the feasibility of isolating exosomes from the plasma of sepsis-induced ALI patients and healthy volunteers.

A label-free technique and a mass spectrometer with consistent quality control were used for proteomic analysis. By researching the combined LC-MS/MS database, we have been able to identify 20,567 peptide-spectrum matches in high confidence, which correspond with 4876 unique peptides. A total of 865 exosome proteins were discovered, with 740 of them being quantified (Supplementary Data 1). The differential proteome dataset used a cutoff of 1.5-fold change and significant *P* values of < 0.05 to identify the altered gene level between healthy donors and sepsis-induced ALI patients. Based on these data, 138 proteins, including APN/CD13, with a ratio higher than 1.5, were classified as being upregulated, while 81 proteins with a ratio of <1/1.5 were deliberated as downregulated, as depicted in a volcano plot (Fig. 1d, Supplementary Data 2). The changes in the quantities of differentially expressed proteins from each group of independent samples were determined using heat maps (Fig. 1e).

Predicting subcellular localization from protein sequences will be useful for predicting protein functions because a protein’s activity is intimately linked to its subcellular localization. Next, we used CELLO v.2.5, (http://cello.life.nctu.edu.tw/) to predict and classify the subcellular structural localization of differentially expressed proteins. As shown in Fig. 1f, the concentration of proteins was observed in the extracellular (44%), cytoplasm (24%), and plasma membrane (15%). We found that the extracellular protein proportion almost reached over half of the total identified protein components, which suggests the important role of secreted proteins in plasma exosomes.

GO annotation was performed for differentially expressed proteins from which, most of them were analyzed for biological process, cellular component, and molecular function were cellular process, extracellular region, and binding, respectively (Fig. 1g). To understand the biological function and key pathways of some differentially regulated proteins in exosomes altered in patients with sepsis-induced ALI, pathway enrichment analysis was performed. KEGG pathway analysis showed the proteins were significantly enriched in protein digestion and relaxin signaling pathway (Fig. 1h).

### Elevated plasma exosomal APN/CD13 level is correlated with increased risk of sepsis-induced ALI

**Fig. 2:**
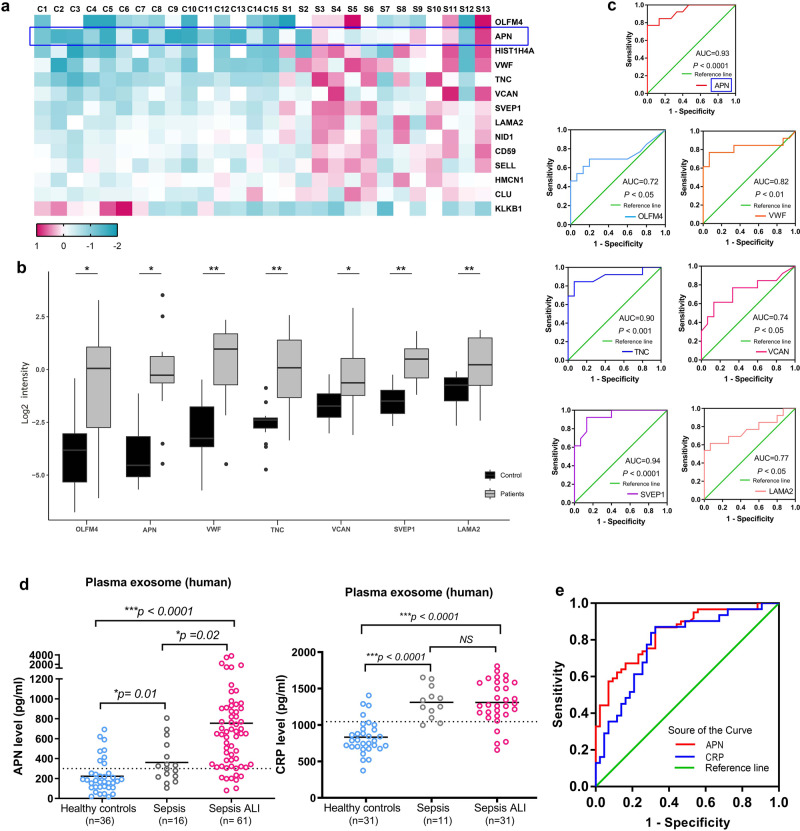
PRM and ELISA verification of the top candidates for differentially expressed proteins. **a** Heatmap display showing 14 differentially expressed proteins from each group of the independent samples. **b** Using PRM, a box plot depicts the log2 sum fragment area of discovered peptides from seven targeted proteins. *P < 0.05, ** *P* < 0.01. **c** The Log2 summed intensity values for peptides identified with PRM for each specified protein were used in ROC analysis to compare the patients to the controls. **d** Compared with individuals in healthy control and sepsis patients, plasma exosomal APN and CRP were expressively enhanced in patients with sepsis-induced ALI (*P* < 0.0001). **e** According to the ROC curve of plasma exosome biomarkers, APN has a significant predictive ability of sepsis-induced ALI: AUC^APN^ > AUC^CRP^ (0.839 vs. 0.781). ROC, receiver operating characteristic. AUC, area under the curve.

To verify the expression of differential proteins in proteomics, we further expanded the sample of plasma exosomes from sepsis-induced ALI patients (*n* = 13) and the control group (*n* = 15) as cohort 2 (Supplementary Table 2) using targeted parallel reaction monitoring (PRM) mass spectrometry and confirmed 14 proteins that were significantly highly expressed in sepsis ALI, as observed in LC-MS/MS analysis. The significantly differentially expressed proteins between the LC-MS/MS and PRM patient cohorts were compared and found to be in good agreement between the two techniques (Table 1 and Supplementary Data 3). Of the 14 proteins, seven candidate proteins were significantly differentially expressed in APN, OLFM4, VWF, TNC, VCAN, SVEP1, and LAMA2 (Fig. 2a, b). To determine the sensitivity and specificity of each protein in connection to sepsis-induced ALI, a ROC analysis was performed, and the area under the ROC curve (AUC) for APN was 0.93 (Fig. 2c), confirming the findings of the differential expression study.

**Table 1:** The differentially expressed proteins between the LC-MS/MS and PRM patient cohorts.

Protein accession	Protein	Sepsis/control Ratio (TMT)	Sepsis/control *P* value (TMT)	Sepsis/control Ratio (PRM)	Sepsis/control *P* value (PRM)
Q6UX06	OLFM4	9.53	2.48E−02	19.59	3.72E−02
P15144	APN	3.42	3.74E−02	17.92	2.77E−02
P62805	HIST1H4A	5.63	1.72E−03	10.31	1.38E−03
P04275	VWF	3.18	2.56E−03	10.08	4.99E−04
P24821	TNC	5.04	5.08E−03	9.87	1.92E−03
P13611	VCAN	4.58	1.30E−02	5.65	3.51E−02
Q4LDE5	SVEP1	3.31	3.24E−03	4.37	1.22E−04
P24043	LAMA2	3.30	6.72E−03	2.88	3.43E−03
P14543	NID1	3.23	2.38E−03	2.61	3.41E−03
P13987	CD59	2.06	3.72E−03	2.18	7.25E−03
P14151	SELL	2.38	2.95E−02	2.13	1.00E−02
Q96RW7	HMCN1	2.53	2.56E−03	1.74	1.70E−02
P10909	CLU	2.06	1.65E−04	1.69	3.45E−02
P03952	KLKB1	0.23	1.36E−03	0.12	3.99E−02
P02741	CRP	8.035	1.59E−04

Furthermore, the proteins were chosen for validation using ELISA, which was performed using the exosomes isolated from individual equal plasma volume samples from cohort 3 (Supplementary Table 3). A total of 61 sepsis-induced ALI, 16 sepsis alone patients, and 36 healthy blood donors were recruited for comparison. The results further confirmed significantly higher levels of APN in plasma exosome samples from sepsis-induced ALI patients than in sepsis alone and the control subjects (Fig. 2d). Furthermore, the degree of differential expression found for these proteins was equivalent to that of the well-known infection biomarker, C-reactive protein (CRP), which is widely utilized in clinical settings as a diagnostic marker of inflammation^24^. Subsequently, we performed ROC curve study to evaluate the specificity and sensitivity of each protein in relation to sepsis-induced ALI patients (Fig. 2e). Next, the predictive performances of exosomal APN and CRP were compared. Our data showed the larger AUC ROC for exosomal APN was 0.839 (95% confidence interval [CI], 0.764–0.914), than that of the CRP at 0.781 (95% CI, 0.674–0.888) (Table 2). This result demonstrates the accuracy of the prediction model with a high level of credibility (a C-index of 1 indicates 100% predictive accuracy). Intriguingly, our data further suggest that higher levels of APN in plasma exosomes are closely associated with sepsis-induced ALI.

**Table 2:** The predictive performances of exosomal APN and CRP compared.

Parameters	AUC (95%CI)	Cut-off value	Sensitivity	Specificity
Exosomal APN	0.839 (0.764–0.914)	300.1 (pg/ml)	86.9 %	69.2%
Exosomal CRP	0.781 (0.674–0.888)	1045.0(pg/ml)	87.1%	67.4%

### Exosomal APN/CD13 is highly expressed in vivo and in vitro, and c-Myc directly induces APN/CD13 expression via binding to its promoter region

**Fig. 3:**
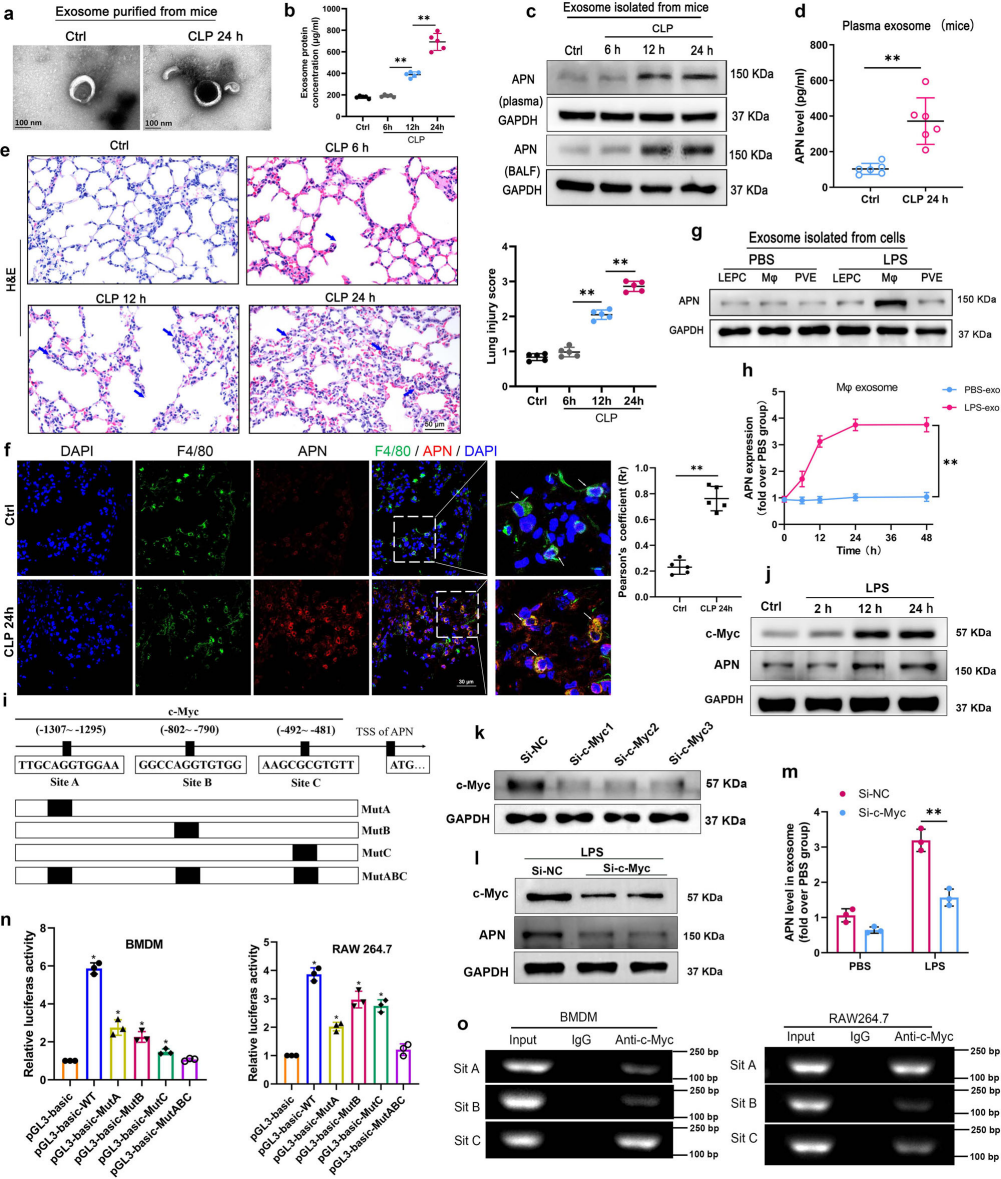
Exosomal APN/CD13 released from macrophages increased significantly in the plasma of septic mice, and c-Myc transcriptionally promotes APN/CD13 expression. **a** Morphology of plasma exosomes isolated from normal and septic mice using TEM. Scale bar, 100 nm. **b** The exosomes were isolated from equal plasma volumes harvested from normal and septic mice to determine the dynamic change in exosome protein concentrations (*n* = 5/group at each time point). **c** The level of APN protein in exosomes was determined using a western blot loaded with equal amounts of exosome protein (30 μg). **d** The levels of plasma exosomal APN were detected using ELISA (*n* = 6/group). **e** Representative H&E staining images of mice lung tissues and lung injury score. The arrows showed alveolar and interstitial edema in H&E. Scale bar, 50 μm. **f** The enlarged double immunofluorescence of the boxed area shows APN (red) deposited in the infiltrating macrophages with F4/80-positive (green). Scale bars, 30 μm. **g** The levels of APN expression in exosomes isolated from macrophage culture supernatant, lung epithelial cells, and pulmonary vascular endothelial cells after 24 h of exposure to 1 μg/mL LPS or PBS as the control. **h** ELISA assay showed that the levels of the exosomal APN from macrophages after stimulation with PBS or 1 μg/mL LPS at different time points. **i** APN promoter regions with possible c-Myc binding sites and the structure of WT (wild type) and mutant binding sites (MutA, MutB, MutC, and MutABC) are depicted in this diagram. **j** Western blotting showing c-Myc and APN expression in BMDMs after 1 μg/mL LPS exposure at different time points. **k** Knocking down c-Myc expression by siRNA significantly attenuated c-Myc expression in BMDMs. **l** c-Myc knockdown also decreased APN levels after LPS exposure in cells and (**m**) in exosomes. **n** The APN promoter’s luciferase activities in BMDM and RAW264.7 cells were transfected with the c-Myc plasmid were identified using a dual-luciferase reporter assay. One-way ANOVA and Dunnett’s multiple comparison test. Mean ± SD, *P < 0.05. **o** PCR gel demonstrating amplification of c-Myc-binding sites A, B, and C following a ChIP assay with anti-c-Myc antibodies. As a negative control, IgG antibody was used.

We isolated the exosomes from the plasma and BALF of mice that had been given CLP to induce sepsis. The sizes of the isolated exosomes were then measured using an electronic microscope (Fig. 3a). In comparison to the control group, the protein concentration of exosomes derived from the plasma of mice significantly increased after CLP from 12 to 24 h (Fig. 3b). Simultaneously, western blot and ELISA revealed that APN expression levels increased in exosomes extracted from septic mice’s plasma and BALF (Fig. 3c, d). Furthermore, we performed H&E staining to examine the extent of lung tissue damage, and the lung injury score was significantly higher with the progression of sepsis (Fig. 3e). The results confirmed the increased levels of APN in the plasma exosomes of septic ALI mice. Subsequently, the histological examination also revealed that APN/CD13 increased in the lung tissues, a significant rise in the macrophages infiltrated into the injured alveolar space at 24 h after CLP. Double immunofluorescence staining showed that APN/CD13 was partially localized in the macrophages (Fig. 3f).

Exosomes contain proteins originating from a specific cell that can be released into the plasma. They are then taken up by distant cells, where they can affect cell function and behavior. To determine the origin of plasma exosomes APN, the culture supernatants of LPS-treated or untreated lung epithelial cells, pulmonary vascular endothelial cells, and macrophages were collected. Exosomes were purified from these supernatants and identified via TEM and NTA (Supplementary Fig. 1). Furthermore, western blot showed a substantial increase in APN expression in the exosomes of LPS-treated macrophages (Fig. 3g). Sustained induction of exosomal APN was observed after exposure to LPS-treated macrophages but not in PBS (Fig. 3h). UCSC, PROMO, and JASPAR bioinformatics software were used to examine a 2 kb region upstream of the APN transcription start site to learn more about the transcriptional regulatory mechanisms that control APN expression. Three c-Myc-binding motifs were identified at −1307 to −1295, −802 to −790, −492 to −481 in the putative APN promoter region. A, B, and C are the names given to the three transcription factor-binding sites (TFBSs) (Fig. 3i). We found that c-Myc and APN protein levels significantly increased in BMDMs after treatment with 1 μg/mL LPS for 12 and 24 h (Fig. 3j). To examine the c-Myc role in APN regulation, we employed small-interfering RNAs to knock down c-Myc expression in BMDM (Fig. 3k). We detected that c-Myc knockdown decreased APN levels following LPS exposure (Fig. 3l). Exosomes were then isolated from BMDM culture supernatant, and the expression level of APN markedly decreased in the LPS-treated si-c-Myc cell-derived exosomes (Fig. 3m). Taken together, these data suggest c-Myc as an upstream regulator of APN.

We first used luciferase reporter assays to see if the c-Myc protein binds to the three sites in the APN promoter. Upregulation of c-Myc in the RAW264.7 and BMDM cell lines increased the activity of the wild-type APN promoter-luciferase. When sites A, B, or C were mutated, a comparable impact was detected, but this effect was not observed when sites A, B, and C were mutated (Fig. 3n). In RAW264.7 and BMDM cells, chromatin immunoprecipitation (ChIP) tests indicated that the c-Myc protein was bound to all three binding sites in the putative APN promoter (Fig. 3o). These findings suggest that c-Myc regulates transcription through binding to the TFBS promoter of APN. Our findings imply that c-Myc increases APN release into exosomes by directly inducing APN expression in macrophages.

### Macrophage-derived exosomal APN/CD13 promote lung epithelial cells RIPK1/RIPK3 necroptosis via increased ROS production and NF-κB signaling activation

**Fig. 4:**
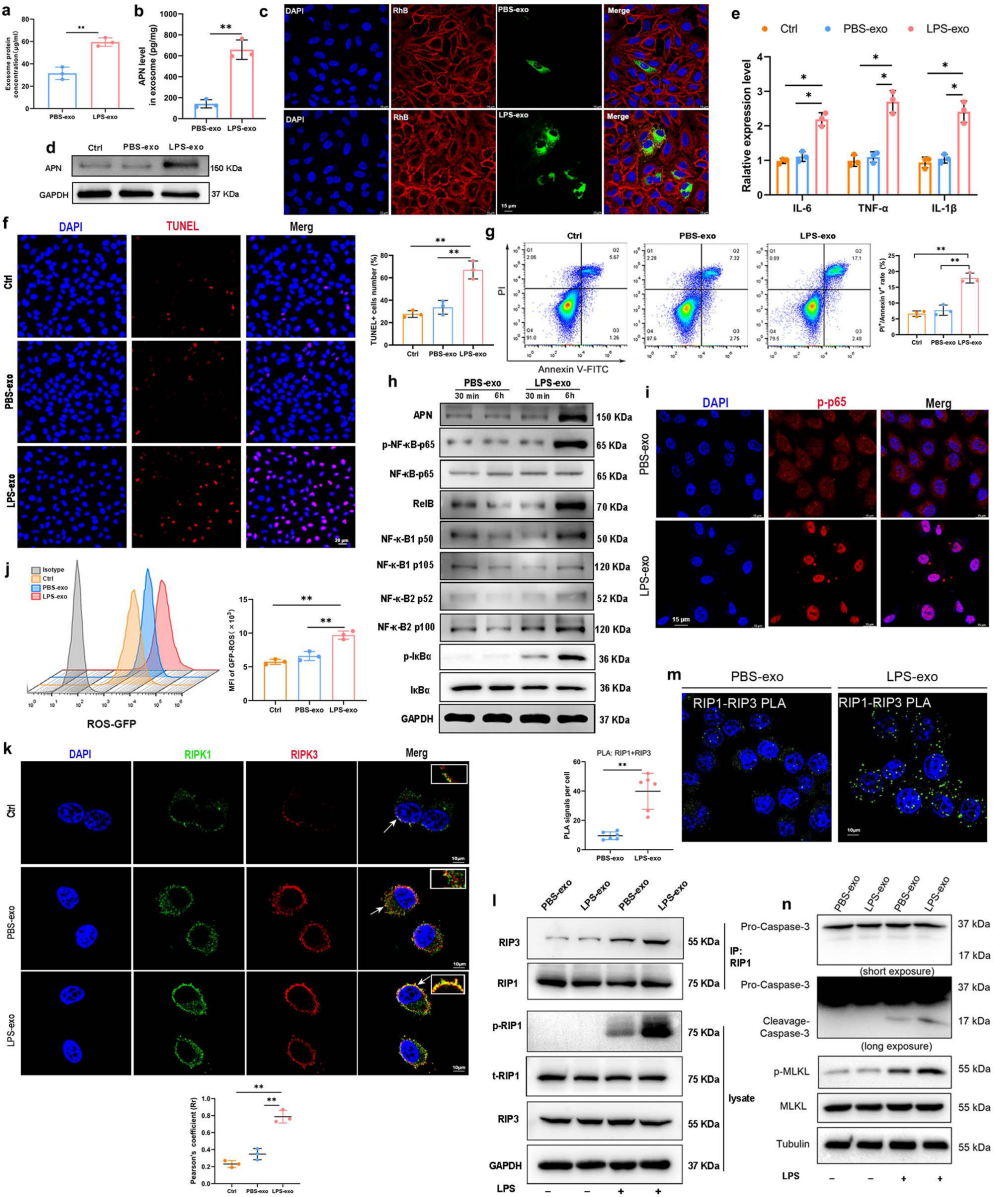
Macrophage-derived exosomal APN/CD13 promotes lung epithelial cell necroptosis. **a** Protein quantification of exosomes secreted by BMDMs exposed to LPS (1 µg/mL) for 24 h using BCA, PBS was used as the control. **b** ELISA was used to determine the levels of APN expression in exosomes. **c** Representative fluorescence images showed LPS-treated BEAS-2B cells incubated with PKH67-labeled exosomes (green) for 6 h. The nuclei were stained with DAPI (blue), and the cytoskeleton with phalloidin (red). Scale bar, 15 μm. **d**–**l** BEAS-2B cells treated with LPS were cocultured for 24 h with exosomes isolated from the supernatant of BMDM stimulated with PBS (PBS-exo) or LPS (LPS-exo). **d** Western blotting was used to detect APN expression in recipient BEAS-2B cells. As an internal reference, GAPDH was used. **e** Effects of BMDM exosomes on the mRNA expression of IL-6, TNF-α and IL-1β in LPS-stimulated BEAS-2B cells. RNA was isolated and analyzed using qPCR. **f** Representative images and quantified data showing BEAS-2B cell apoptosis determined by a percentage of terminal deoxynucleotidyl transferase-mediated dUTP nick-end labeling (TUNEL)-positive nuclei/total nuclei in the indicated group (*n* = 3). Scale bar: 20 μm. **g** Flow cytometric analysis shows the proportion of apoptotic cells (Annexin V/PI-positive), after treatment with BMDM-derived exosomes. Bar graphs depict the quantitative analysis. **h** Western blotting analysis of the protein levels of NF-κB signaling genes shows that the levels of P-p65, RelB, p50, and p100 were significantly elevated in the recipient BEAS-2B cells treated with BMDM-derived exosomes in the presence of LPS. The BEAS-2B cells were co-treated with exosomes and LPS (1 μg/mL) for 30 min or 6 h. **i** Immunofluorescence was used to examine the translocation of the NF-κB p65 subunit from the cytoplasm to the nucleus. The blue spots are cell nuclei, and the red spots are NF-κB p65 staining. **j** Flow cytometry analysis of intracellular ROS level. **k** The increase in RIPK1-RIPK3 complex in BEAS-2B cells after co-treatment with LPS-exo, indicated by immunofluorescence and (**l**) immune-precipitation. **m** Endogenous RIP1 interacts with RIP3 indicate necrosomes formation by treatment with LPS-exo or PBS-exo. Cells were fixed and proximity ligation assay (PLA) was performed using α-RIP1 and either α-RIP3, as indicated. The signal from each pair of PLA probes is visualized as an individual fluorescent spot. Representative confocal microscopy images are shown for each condition, which was repeated at least three times. Scale bar, 10 μm. PLA signals per cell were quantified by image software-assisted analysis. **n** Western blotting analysis of the protein levels of caspase-3 cleavage and p-MLKL. Ctrl, LPS-treated BEAS-2B cells without exosomes. All of the results are based on three separate experiments. The data are presented as the mean ± SD (*n* = 3), *P < 0.05, **P < 0.01.

Exosomes are capable of carrying functional proteins to modulate the functions of recipient cells. To observe the transfer of exosomes from macrophages to lung epithelial cells, we performed coculture experiments. First, purified exosomes were separated from the culture supernatants of BMDMs treated with PBS and 1 µg/mL LPS for 24 h. We found a higher number of exosomes in LPS-treated BMDMs (LPS-exo) than in PBS-treated (PBS-exo) BMDMs, as shown using BCA analysis (Fig. 4a). This was supported by a significant rise in APN expression in the LPS-exo group (Fig. 4b). The uptake of macrophage exosomes by lung epithelial cells was then investigated in vitro. We labeled the exosomes with PKH67 and added them to LPS-stimulated BEAS-2B cells for 6 h. An increase in the internalization of PKH67-labeled LPS-exo was detected in LPS-injured BEAS-2B cells (Fig. 4c). When BEAS-2B cells were treated with LPS-exo, the level of APN increased considerably (Fig. 4d). These results indicated that APN was loaded into exosomes and transferred to recipient lung epithelial cells in the presence of LPS.

For the pro-inflammatory cytokines, the levels of IL-6, TNF-α and IL-1β were elevated in cells cocultured with LPS-exo (Fig. 4e). In addition, LPS-exo markedly induced apoptosis of BEAS-2B cells in the presence of LPS, as observed using TUNEL staining (Fig. 4f). Flow cytometric analysis also showed that LPS-exo significantly increased the proportion of PI^+^/Annexin V^+^ cells which confirmed that BEAS-2B cells co-cultured with LPS-exo for 6h showed significantly increased apoptosis as compared to BEAS-2B cells co-cultured with PBS-exo (Fig. 4g). As shown in Fig. 4h, to confirm whether exosomal APN was transferred to BEAS-2B cells under the influence of LPS, we measured APN expression in BEAS-2B cells after being cocultured with LPS-exo following LPS treatment for 6 h. Meanwhile, we found that the levels of NF-κB signaling genes, including p-p65, RelB, p50, p100, and p-IκBα were significantly elevated in recipient cells. LPS-exo caused an increase in nuclear translocation of NF-κB p65, as revealed by immunofluorescence results (Fig. 4i). Thus, we concluded that exosomal APN affected the activation of NF-κB signaling.

Figure 4j showed that intracellular ROS levels significantly increased when cocultured with LPS-stimulated BMDM exosomes. Furthermore, after coculture with LPS-stimulated BMDM exosomes, the amount of the RIPK1-RIPK3 complex, the typical marker of necroptosis, increased dramatically over time (Fig. 4k, l). Consistently, we performed a proximity ligation assay (PLA) to verify direct binding of endogenous RIP1 and RIP3 indicated necrosomes formation by treatment with LPS-exo (Fig. 4m). Next, cleaved caspase 3 for apoptosis and specific marker proteins p-MLKL for necroptosis were detected by western blot. As expected, the protein levels of cleaved caspase-3 and p-MLKL were increased in the LPS-exo treated BEAS-2B cells (Fig. 4n). These data provide compelling evidence that LPS-treated macrophage-derived exosomes, which transfer APN into lung epithelial cells, can promote necroptosis.

### Macrophages derived exosomal APN/CD13 leads to increased mitochondrial dysfunction and activating NF-κB signaling by binding to receptor TLR4

**Fig. 5:**
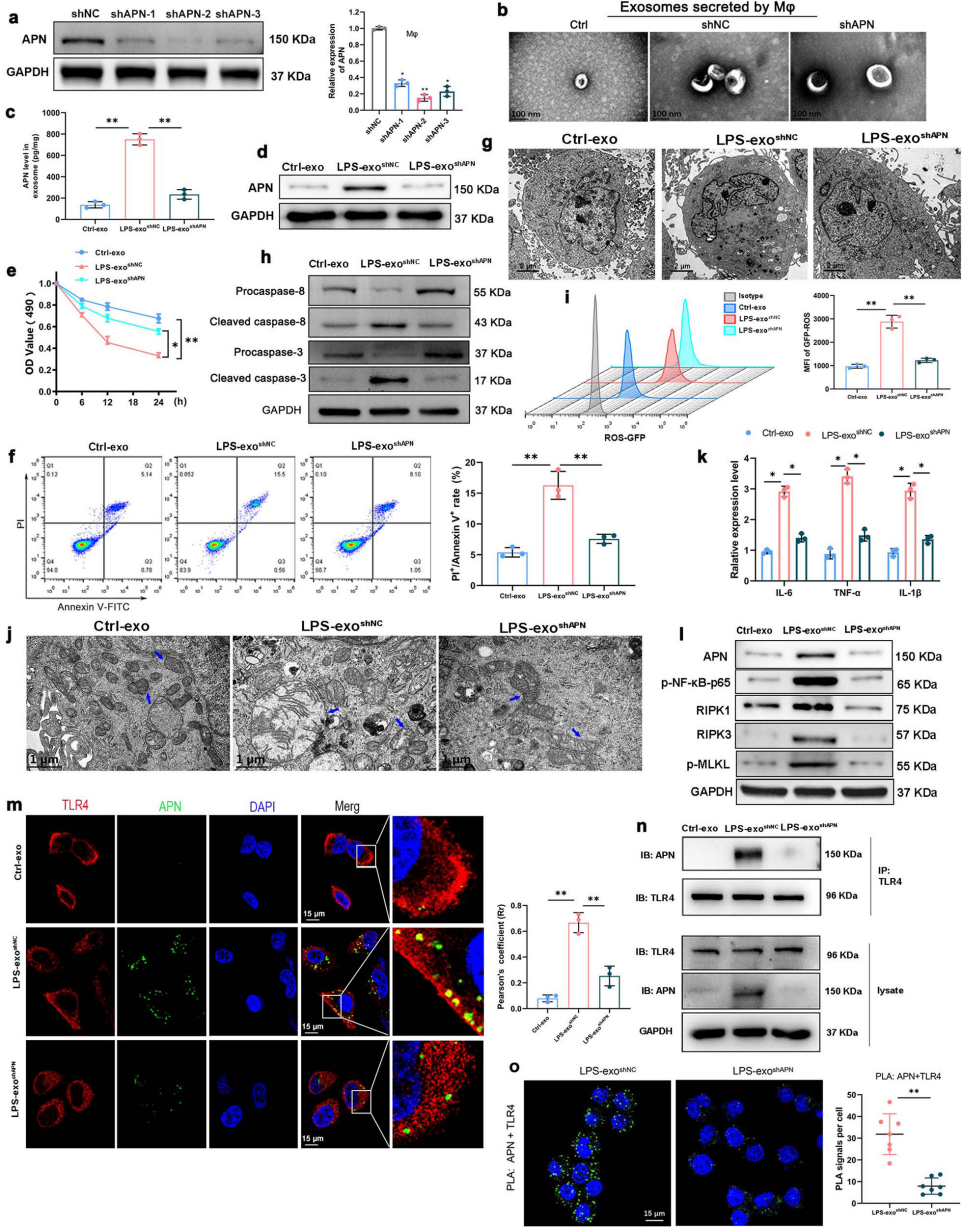
Macrophage-derived exosomal APN/CD13 promotes lung epithelial cell necroptosis by binding to the receptor TLR4. **a** The APN expression levels were measured using western blotting and qRT-PCR in macrophages treated with LV-shNC or LV-APN. **b** Isolated exosomes under the electron microscope. Scale bar: 100 nm. **c** ELISA detection of APN expression in exosomes derived from macrophages transfected with shNC or shAPN in the presence of LPS. Exosomes from PBS-treated macrophages were used as the negative control. Ctrl-exo, exosomes from macrophages without LPS, LPS-exo^shNC^ exosomes from shNC macrophages with LPS treatment, LPS -exo^shAPN^ exosomes from shAPN macrophages with LPS treatment. After lung epithelial cells were cocultured with these exosomes for 24 h in the presence of LPS (1 µg/mL), the expression of APN was measured by western blotting (**d**), the viability of the cells was examined using the CCK8 assay (**e**), analyses of Annexin V/PI staining using flow cytometry (**f**). **g** The characteristic morphological changes in necroptosis in LPS-exo^shNC^-treated BEAS-2B cells were observed using TEM. **h** Western blotting analysis of the expression of apoptosis-related protein cleaved caspase-8 and cleaved caspase-3 in BEAS-2B cells. **i** Measurement of intracellular ROS level via flow cytometry, (**j**) the mitochondrial structures were observed using an electron microscope. **k** RT-PCR analysis of the mRNA expression of IL-6, TNF-α and IL-1β. **l** The expression of p-p65, RIPK1, RIPK3, and p-MLKL was detected using western blotting. **m** Colocalization of APN (green) and TLR4 (red) in LPS-exo^shNC^-treated BEAS-2B cells using confocal microscopy. Scale bar, 50 μm. **n** Cell lysates from BEAS-2B cells precipitated with agarose beads were pulled down and probed for TLR4 and APN protein levels with control IgG or TLR4 mAb. **o** PLA was performed using specific antibodies against APN and TLR4. Nuclei were stained with DAPI. Scale bars represent 15 μm. PLA signals per cell were quantified by Image software-assisted analysis. All results are based on three measurements. The information is presented as the mean ± SD (*n* = 3), *P < 0.05, **P < 0.01.

To investigate whether APN mediates the effects of macrophage exosomes on lung epithelial cell necroptosis, we tried to silence APN expression by designing three short hairpin RNAs (shRNA1, shRNA2, and shRNA3). qRT-PCR and western blotting revealed a fourfold reduction in APN expression in BMDM cells treated with shRNA relative to the control group; shRNA2 was was chosen for future study because it was the most effective shRNA among all (Fig. 5a). Following LPS treatment, the exosomes isolated from shAPN-transfected BMDM cells (LPS-exo ^shAPN^) expressed less APN compared with the exosomes isolated from NC-transfected BMDM cells (LPS-exo ^shNC^) (Fig. 5b, c). Notably, LPS-exo ^shAPN^ or LPS-exo ^shNC^ were administered to BEAS-2B cells treated with LPS, and we observed that inhibition of APN markedly reversed the macrophage exosome-induced upregulation of APN in recipient lung epithelial cells (Fig. 5d). Subsequently, it was found that LPS-exo^shAPN^ significantly alters the decrease in cell viability and increased the proportion of the apoptotic cells in the LPS-exo^shNC^ treatment group (Fig. 5e–g). Consistently, the activity of apoptosis-related proteins caspase-3 and caspase-8 was also measured in BEAS-2B cells. As expected, the protein levels of cleaved caspase-3 and cleaved caspase-8 increased in the LPS-exo^shNC^ treatment group, while it was strongly reversed by LPS-exo^shAPN^ (Fig. 5h).

Furthermore, when LPS-exo^shAPN^ was administered to BEAS-2B cells treated with LPS, we observed a significant decrease in mitochondrial dysfunction and decreased levels of ROS compared to LPS-exo ^shNC^ treatment (Fig. 5i, j). Further studies revealed that pro-inflammatory cytokines IL-6, TNF-α and IL-1β were remarkably decreased in LPS-exo^shAPN^-stimulated cells compared to those stimulated with LPS-exo^shNC^ (Fig. 5k). Consistently, LPS-exo^shNC^ upregulated p-NF-kB p65, RIPK1, RIPK3, and p-MLKL protein levels. Notably, inhibition of APN abrogated the ability of LPS-exosomes in recipient BEAS-2B cells (Fig. 5l).

In addition, confocal imaging confirmed the transport of exosomal APN from macrophages and consistently colocalized with TLR4, which localized on the cell surface of lung epithelial cells (Fig. 5m). Accordingly, TLR4 was found in the APN immunoprecipitate (Fig. 5n) after LPS-exo^shNC^-stimulated immunoprecipitation of BEAS-2B cell lysates, indicating that the two proteins are part of the same protein complex. To confirm that APN/CD13 binds and stimulates TLR4, we performed a PLA, in which positive signals (green immunofluorescent dots) appear only when APN interacts with and therefore is in close proximity to TLR4. The data revealed a direct interaction between APN/CD13 and TLR4 (Fig. 5o). These results indicate that macrophage-derived exosomal APN, by binding to the receptor TLR4 induces ROS production, accelerates mitochondrial dysfunction, and activates NF-κB signaling pathways.

### Exosomal APN/CD13 promotes the inflammatory response and lung tissue injury in vivo

**Fig. 6:**
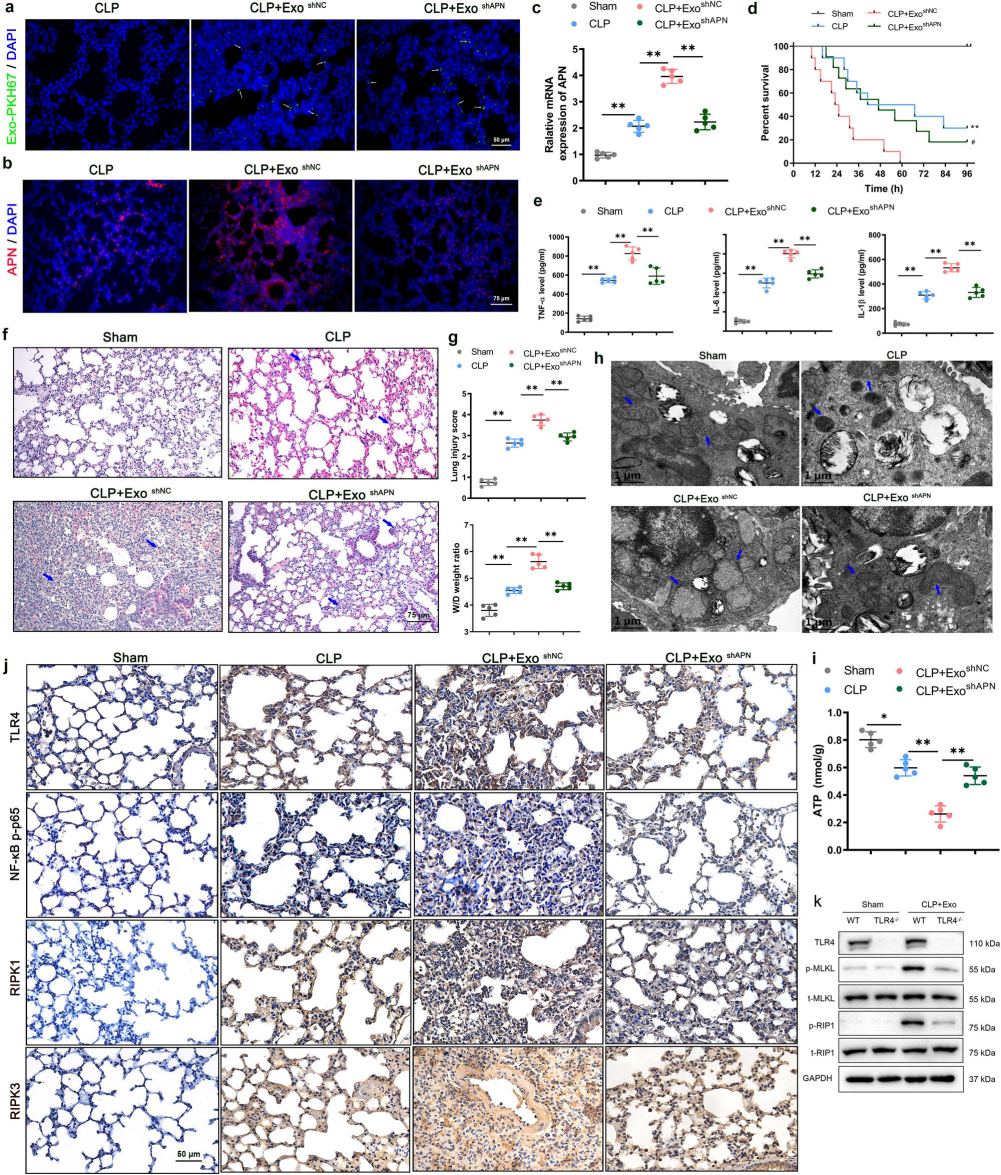
Exosomal APN promotes the lung tissue injury and inflammatory response in CLP model mice. The mice were subjected to sham or CLP and injected with LPS-exo^shNC^ or LPS-exo^shAPN^ (100 μg/mouse) or control PBS (same volume) immediately after CLP surgery. CLP + Exo^shNC^ CLP mice were injected with LPS-exo^shNC^, CLP + Exo^shAPN^ CLP mice were injected with LPS-exo^shAPN^. **a** Fluorescent images showed the accumulation of PKH67-labeled LPS-exo in the lung tissue of septic mice 6 h after injection. Scale bars, 50 µm. **b** Representative immunofluorescence images of APN (red) staining in lung tissues (*n* = 5). Scale bars, 75 µm. The mice were euthanized 24 h after injection. **c** RT-PCR analysis of APN in the lung of LPS-exo^shAPN^ or LPS-exo^shNC^-treated CLP mice. **d** Kaplan–Meier survival analysis of LPS-exo^shAPN^ or LPS-exo^shNC^-treated mice subjected to CLP as described in Materials and Methods for a 96 h survival study (*n* = 10, **P < 0.01 CLP + Exo^shNC^ vs. CLP, ^#^*P* < 0.05 CLP + Exo^shNC^ vs. CLP + Exo^shAPN^) . **e** ELISA was used to measure the levels of pro-inflammatory cytokines IL-6, TNF-α and IL-1β in BALF. CLP + Exo^shNC^ group showed significant upregulation of IL-6, TNF-α and IL-1β, while shAPN reversed the upregulation significantly (*n* = 5). **f** The H&E staining was used to detect lung pathology, (**g**) lung injury score was evaluated due to the pathology, and the levels of water content in the lung tissues were analyzed using the W/D method. **h** Lung mitochondrial structures were observed using an electron microscope (*n* = 5), Scale bars: 1 µm. **i** A bioluminescence-based assay was used to determine the ATP content of lung homogenates. The results are given in nmoles of ATP produced per gram of wet tissue. **j** Representative immunohistochemical images of TLR4, as well as p-p65, RIPK1, and RIPK3 in the lung tissue lesions of the Exo-treated CLP mice and the control groups (*n* = 5). Scale bars: 50 μm. **k** Western blotting analysis of the expression of necroptosis-related protein p-MLKL, MLKL, p-RIP1 and RIP1 in mice lung tissues. All results are based on three measurements. The data are presented as the mean ± SD (*n* = 3), *P < 0.05, **P < 0.01.

To further investigate the potential role of exosomal APN from macrophages in vivo, we intravenously injected septic mice with LPS-exo^shNC^ or LPS-exo^shAPN^ (100 μg/mouse) or control PBS immediately after CLP surgery. The biodistribution of LPS-exo was investigated by labeling exosomes with PKH67. We intravenously injected PKH67-labeled LPS-exo into the CLP mice. The fluorescence levels of the frozen lung section corroborated the robust accumulation of LPS-exo in the lung tissue of septic mice (Fig. 6a). Furthermore, after treatment with LPS-exo^shNC^, the protein level of APN increased significantly in the lung tissues; however, this effect was abrogated in the LPS-exo^shAPN^ CLP group (Fig. 6b, c). The survival of the mice was observed for 4 days (96 h). Compared to mice treated with either LPS-exo^shAPN^ (CLP + Exo^shAPN^) or PBS (CLP), septic mice treated with LPS-exo^shNC^ (CLP + Exo^shNC^) had a significantly lower survival rate. The difference in mortality between the CLP + Exo^shAPN^ and CLP groups was not significant (Fig. 6d). Therefore, exosomal APN deficiency exerts a protective effect, as shown by prolonging the survival of severely septic mice.

We evaluated pro-inflammatory cytokines in the BALF of septic mice to verify the impact of exosome-containing APN on the inflammatory response and lung tissue injury. The CLP + Exo^shNC^ group showed substantial upregulation of pro-inflammatory cytokines IL-6, TNF-α and IL-1β while shAPN-Exo significantly reversed this upregulation (Fig. 6e). The pathological injury of the lung tissues, and the lung wet-to-dry (W/D) ratio and, lung injury score was significantly augmented in CLP+Exo^shNC^ mice compared with those in the CLP + Exo^shAPN^ and CLP groups (Fig. 6f, g). These findings demonstrate that APN can be functionally transported to lung tissue and can cause pulmonary inflammation via macrophage-derived liposomes. As compared to the CLP + Exo^shNC^ group at 24 h after CLP, the CLP + Exo^shAPN^ group showed significantly decreased mitochondrial dysfunction, as evidenced by fewer changes in mitochondrial morphology and significantly higher mitochondrial ATP content (Fig. 6h, i). Moreover, the effect of exosomal APN on TLR4, p-p65, RIPK1, and RIPK3 was also observed using immunohistochemistry in the lung tissues. Compared with the negative control group, LPS-treated macrophage-derived exosomes increased the expression of p-p65, RIPK1, and RIPK3, which positively correlated with APN, while mice treated with shAPN macrophage-derived exosomes showed decreased levels of their mRNA expression (Fig. 6j). Moreover, the pathological injury of the lung tissues was significantly attenuated in TLR4 knockout mice with APN in the purified exosomes compared to wild-type mice (Supplementary Fig. 2). Western analysis showed that the protein levels of necroptosis- related protein p-MLKL and p-RIP1 in the lung tissues of TLR4^−/−^ mice are significantly reduced relative those of wild type mice (Fig. 6k). Taken together, these data show that exosomal APN secreted by macrophages may aggravate sepsis-induced ALI by promoting the inflammatory response and activating the necroptosis pathway.

### Elevated plasma exosomal APN/CD13 level is associated with severity of sepsis-induced ALI patients

We further verified whether plasma exosomal APN/CD13 levels were associated with sepsis ALI outcomes. Patients were classified as survivors or non-survivors. They were equivalent at baseline, with no significant difference between the groups regarding demographics and clinical characteristics (Supplementary Table 4).

**Fig. 7:**
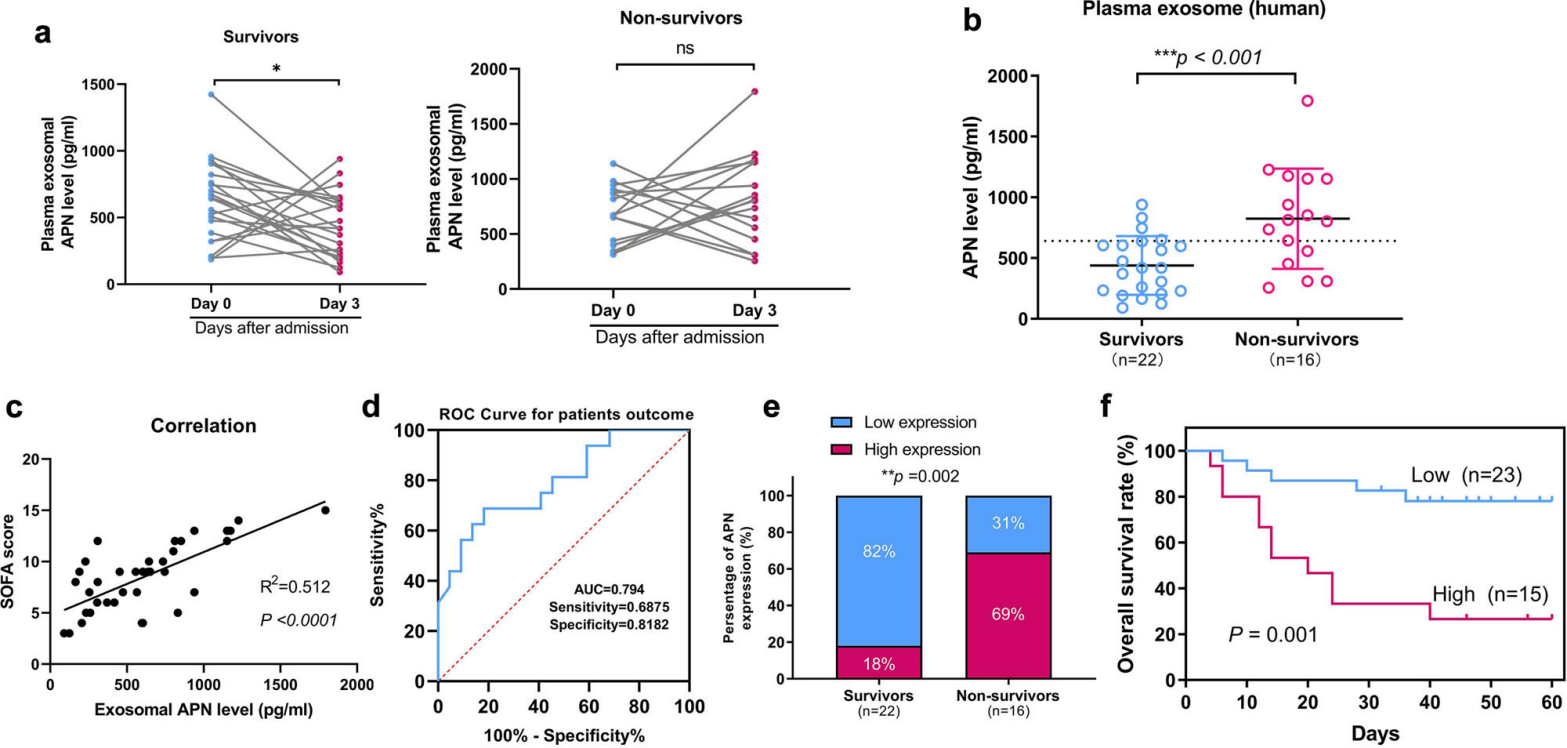
The elevated plasma exosomal APN level is independently related to a higher risk of poor outcomes. **a** Compared with day 0 admission of ICU plasma exosomal APN levels of patients with sepsis-induced ALI decreased at the day 3 in survivors (*P < 0.05, no change observed in non-survivors. **b** APN expression levels in plasma exosomes were higher in non-survivors compared with those in survivors on the day 3. **c** The relation between plasma exosomal APN levels and SOFA score. **d** The ROC curve for individual APN expression level in the plasma exosome of patients on the day 3 to separate good (survivors) outcomes from poor (non-survivors) outcomes during 2 months. **e** The subgroup of high plasma exosomal APN level was more probable to suffer poor outcomes than the subgroup of low exosomal APN after treatment in ICU (69% vs. 31%). **f** A high plasma exosomal APN level correlated significantly with an increased mortality rate in patients with severe sepsis ALI/ARDS for 2 months (*P* = 0.001).

To further explore the changing level of circulating exosomal APN from the day admission to the ICU (day 0) up to the day 3 post-admission to the ICU, blood samples of 38 patients were collected and exosomes were purified from the plasma: the average APN levels in plasma exosomes decreased significantly in survivors from 0 to day 3 with no change in non-survivors (Fig. 7a). To assess the relationship between plasma exosomal APN and sepsis-induced ALI mortality, the measurements were categorized into survivors and non-survivors on the day 3 post-admission to the ICU. Plasma exosomal APN levels were higher in the non-survivors and positively related to SOFA score (Fig. 7b, c). Furthermore, we created a ROC curve for plasma exosomal APN expression based on the outcome to assess the efficacy of plasma exosomal APN expression in predicting the prognosis of sepsis-induced ALI. Our data showed that the AUC ROC curve for APN was 0.794 (95% CI, 0.649–0.939) (Fig. 7d). ROC curves and Youden’s index were used to establish the cutoff value. (Youden’s index = sensitivity + specificity − 1). According to Youden’s index, a cutoff of 641.1 pg/mL had the best sensitivity (68.8%) and specificity (81.8%) for distinguishing patients with bad results from those with favorable outcomes. Survivors were observed in 4 of 22 (18%) patients with high plasma exosomal APN levels (≥641.1 pg/mL), while only 5 of 16 (31.25%) patients with low plasma exosomal APN levels (<641.1 pg/mL) were observed in non-survivors (Fig. 7e). In addition, survival analysis further indicated that elevated plasma exosomal APN expression levels remained significantly correlated with poor prognosis in patients with severe sepsis ALI/ARDS. (Fig. 7f). Together, these data suggest that higher plasma exosomal APN levels are associated with poor prognosis.

## Discussion

**Fig. 8:**
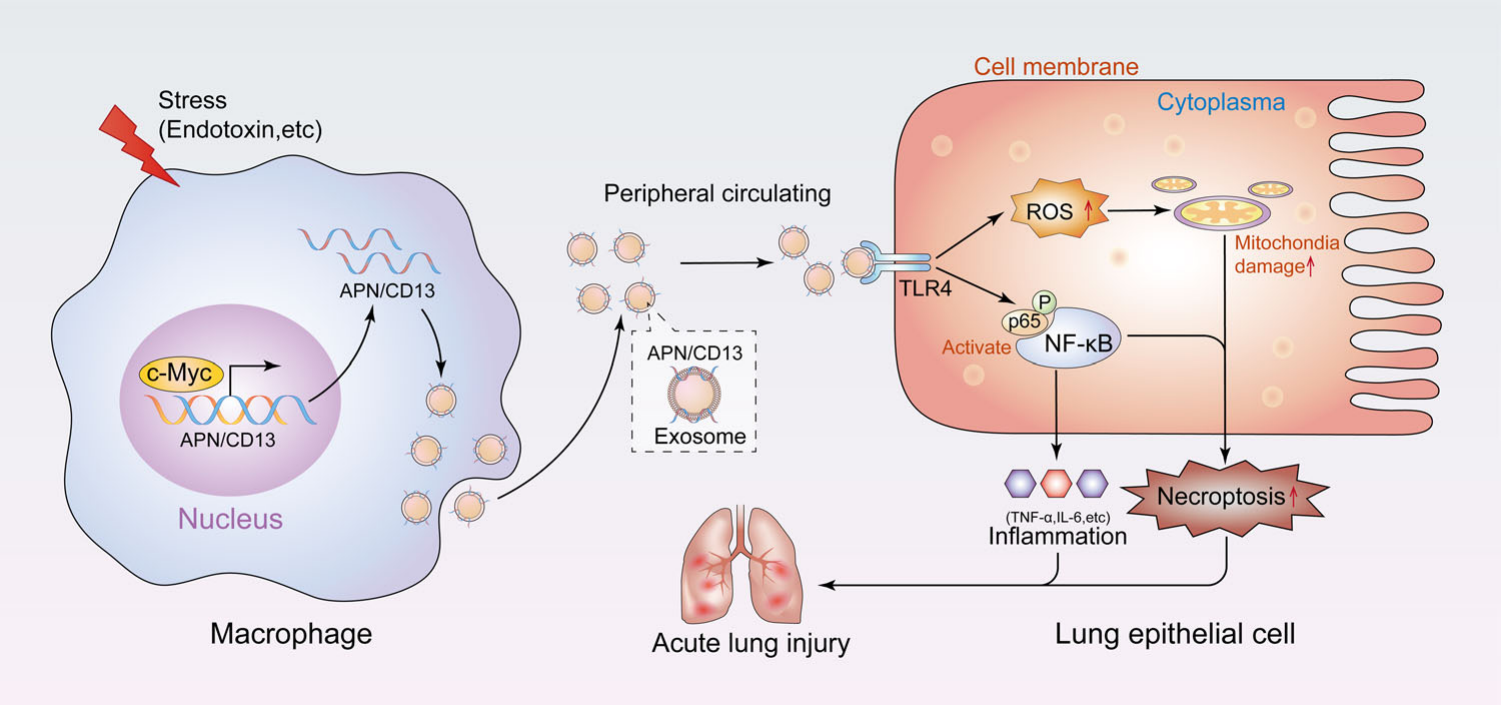
Working model. Our data demonstrated that c-Myc directly induces APN/CD13 expression in LPS-stimulated macrophages, which promotes APN/CD13 secretion into exosomes. In addition, we found that exosomal APN/CD13 from macrophages regulates the necroptosis of epithelial cells by binding to the cell surface receptor TLR4, enhancing NF-κB activity and mitochondrial dysfunction, and promoting RIPK1/RIPK3 necroptosis. In conclusion, our findings reveal that the communication between macrophages and lung epithelial cells via exosomal APN/CD13 leads to mitochondrial dysfunction, augments inflammation and increases cell apoptosis, so that participates in the occurrence of sepsis-induced ALI.

In this study, we found increased levels of exosomal APN/CD13 in the plasma of the patients who suffered from septic ALI and a similar increase occurred in septic mice. Moreover, c-Myc directly induces APN/CD13 expression in LPS-stimulated macrophages, which promotes APN/CD13 secretion into exosomes. We found that exosomal APN/CD13 did not only significantly accelerate LEPC necroptosis in vitro but also promoted the inflammatory response and lung tissue injury in vivo. Next, the mechanism through which exosomal APN/CD13 regulated LEPC necroptosis was further investigated by demonstrating how it binds to the receptor TLR4, which is localized on the cell surface of epithelial cells, induces ROS production, accelerates mitochondrial dysfunction, and activates NF-κB signaling pathways (Fig. 8). Our findings provide a novel molecular target for the treatment of sepsis-induced ALI.

Of the 14 proteins confirmed using PRM, we validated seven proteins (APN, OLFM4, VWF, TNC, VCAN, SVEP1, and LAMA2) with diagnostic potential for the detection of sepsis-induced ALI (Fig. 2). Three of these proteins, especially APN, were remarkably upregulated in the exosomes of patients. Consistent with the results of previous studies, plasma von Willebrand factor antigen (VWF) levels were significantly higher in ALI/ARDS sepsis patients, and the plasma level of VWF was independently related to mortality^25–27^. Previous studies have identified exosomal miRNAs as valuable biomarkers of ALI/ARDS^10^. However, the excretion of proteins in plasma exosomes from patients with sepsis has not been examined in ALI. Exosomal proteins in plasma were shown to have a significantly distinct profile in this investigation, indicating that exosomal APN/CD13 may play a potential role in ALI.

In addition, we further verified that elevated plasma exosomal APN levels were associated with the ALI severity and mortality prediction in a small single-center study of 38 patients. Interestingly, recent studies have also indicated that CD13 positivity is an independent poor prognostic indicator of survival and recurrence in HCC^28^. Our study revealed that plasma exosomal APN may help to predict the prognosis of sepsis and ALI patients. These data agree with a recently published clinical study of 220 patients, which indicated that enhanced plasma level exosome was linked with severe organ failure in critically ill sepsis patients^29^. Furthermore, it has shown that the biological effects of exosomes on recipient cells are largely reliant on the miRNAs or loaded proteins^30^. Therefore, our study may provide novel diagnostic and prognostic biomarkers for sepsis by acquiring proteomic profiles of plasma exosomes.

Although APN is a relatively known molecule, our findings on its role in sepsis-induced ALI are novel. To determine the origin of plasma exosomal APN, we examined the exosomes from the culture supernatant of cells and found a remarkably high expression of APN in exosomes of macrophage repolarization from M1 (LPS-stimulated). Exosomes derived from macrophages account for a large proportion of circulating microvesicles in the blood^31^. Previous research has shown that LPS stimulation of macrophages releases exosomes that involve chemokines or miRNAs, leading to an inflammatory response and tissue damage^32,33^. In contrast, HIV-infected macrophage-secreted exosomal miR-27a mediates lung epithelial cell dysfunction^34^. These data suggested that APN is an important effector of exosomes secreted by LPS-stimulated macrophages and participates in sepsis-induced ALI.

LPS stimulation of macrophages increased the level of CD13 protein by more than fourfold in a time-dependent manner^35^. Consistent with these findings, we observed that the levels of APN/CD13 and c-Myc increased in LPS-stimulated macrophages. However, c-Myc knockdown significantly attenuated APN expression in macrophages and decreased APN levels in exosomes after LPS exposure. Interestingly, a previous study found c-Myc transcriptional regulation of a series of genes in macrophages^36^. c-Myc is a key transcription factor involved in cell growth, apoptosis, survival, and metastasis^37,38^. In our study, we identified a region upstream of the APN/CD13 locus revealing various putative binding sites for c-MYC. Subsequent experiments authenticated that c-Myc positively regulates the biogenesis of APN/CD13 via direct binding to its promoter region. These results confirm that c-Myc directly induces APN/CD13 expression in macrophages and promotes the APN expression in exosomes secreted by macrophages.

Exosomes can deliver their content into recipient cells by modifying their physiological state^39^. Epithelial cell damage caused by circulating exosomes may contribute to multiple organ dysfunction in sepsis^40,41^. Interestingly, we observed that the lungs could take up circulating exosomes, and exosomal APN deletion significantly prolonged the short-term survival of septic mice. The rise in APN could be related to the co-effect of endotoxin and exosome transport in LEPC, which promotes necroptosis, as both exosomes and endotoxin are present in the circulation throughout sepsis. During ALI, multiple cell death causes, including apoptosis and regulated necrosis, contribute to lung injury^42^. Furthermore, there is strong evidence regarding the involvement of exosomes in the ALI development^43^, and it has been demonstrated that the incorporation of blood exosomes from LPS-treated rats into naive rats generates ARDS^44^. To our knowledge, the detailed mechanisms of exosomal APN-induced LEPC necroptosis have not been previously documented.

Necroptosis is a kind of cell death that controls the amount of tissue degradation and, as a result, organ function^45^. Next, the mechanism through which exosomal APN regulated LEPC necroptosis was further investigated by demonstrating its acceleration of mitochondrial dysfunction and regulation of the NF-κB signaling pathway. Previous research has revealed the direct action of CD13 on intracellular signaling pathways via involvement of auxiliary proteins^16^, or through the organization of plasma membrane, it can directly act on cell signaling, and modulate cellular localization/activation of cytokine receptors^46^. We observed that exosomal APN could bind to the receptor TLR4, which is localized on the cell surface of LEPC. In addition, another study^35^ found that TLR4 and CD13 are present in a protein complex, and CD13 modulates TLR4 receptor activation of NF-κB and induces ROS production. The activation of NF-κB is regularly the dominant response to death receptors^47,48^. ROS contribute to the regulation of necroptosis, and the function of ROS in necroptosis is to enhance RIP3-containing complex necrosome formation^49^. Moreover, programmed necrosis can be triggered via the TLR4 pathway^50^. Based on previous evidence, we propose that exosomal APN/CD13 promoting LEPC necroptosis may be associated with its activation of the NF-κB signaling pathway, and mitochondrial dysfunction exacerbated the production of ROS. Recent research has also demonstrated that CD13 prevents the degradation of p65 and activates the NF-κB signaling pathway^28^. We demonstrated that macrophage-excretive exosomal APN was capable of triggering LEPC necroptosis via the APN-associated mechanism, providing new insight into the mechanism of LEPC functional disorder in sepsis-induced ALI.

Interestingly, we found a significant increase in plasma exosomal APN levels in non-survivors than in survivors. Importantly, plasma exosomes were demonstrated to originate partly from macrophages, and we observed the positive correlation of expression of plasma exosomal APN/CD13 with the severity of sepsis. In sepsis, patients with elevated plasma levels of exosomal APN/CD13 had a poor prognosis. In comparison to previous research, our findings clearly demonstrated that the overall plasma levels of exosomal APN/CD13 in sepsis patients were associated with ALI and mortality, implying its potential as a biomarker to assess the severity and mortality in sepsis. However, more multicenter studies with larger sample sizes are needed to confirm its efficacy and reliability as a sepsis biomarker. Thus, these clinical data confirm that macrophage exosomal APN/CD13 contributed to LEPC necrosis by demonstrating its acceleration of mitochondrial dysfunction and regulation of the NF-κB signaling pathways and may serve as a potential biomarker of sepsis ALI.

Finally, we found that high plasma exosomal APN levels are associated with sepsis-induced ALI and predict a high risk of poor outcomes in septic patients. LPS-stimulated macrophages and c-Myc transcriptional regulation induce the expression of APN/CD13, which promotes exosomal APN/CD13 secretion in the peripheral blood. Furthermore, plasma exosomal APN/CD13 promotes epithelial cell RIPK1/RIPK3 necroptosis by binding to TLR4 receptors, which are localized on the cell surface of epithelial cells, inducing ROS production, accelerating mitochondrial dysfunction, and activating the NF-κB signaling pathways. The present study tells the significance of circulating plasma exosomes in regulating information transfer between macrophages and lung epithelial cells during sepsis. This new intercellular communication pathway could help researchers to better understand how ALI develops in response to diverse stressors, such as sepsis. These findings imply that exosomal APN/CD13 could be a new therapeutic target for sepsis-induced ALI progression diagnosis and treatment.

## Materials and methods

### Patient recruitment and sample collection

The study was approved by the Medical Ethics Committee of the Shenzhen Hospital of Southern Medical University (Shenzhen, China, approval ID: NYSZYYEC20200039). Clinical data have been registered in the China Clinical Trials Registry (NO. ChiCTR2100043761). Healthy volunteers were recruited from the hospital personnel and through advertisements. From November 1, 2018 to December 31, 2020, all patients were admitted to the ICU of the Shenzhen Hospital of Southern Medical University. The Third International Consensus Definitions for Sepsis and Septic Shock (Sepsis-3) were used to diagnose sepsis. During the same period, we also found patients who met the American-European Consensus Conference criteria for ALI. The APACHE II and SOFA scores were used to determine the severity of the sickness. According to institutional rules, patients were treated by the physicians working in ICU who were not involved in the study. The supplementary data contains further information.

Blood samples (10 mL) were collected from healthy subjects and patients with sepsis-induced ALI using venipuncture, and plasma was separated using 10 min centrifugation at 1200 × *g* and stored at −80 °C for further use.

### Isolation, characterization, labeling, and uptake of exosome

According to a previously established approach, exosomes were extracted using a modified differential ultracentrifugation protocol (Beckman Coulter). The first stage involves the removal of big cell fragments or debris via centrifugation at 3000 *g* for 10 min at 4 °C. the remaining smaller cellular debris in the supernatant was further removed by centrifuged at 10,000 × *g* for 30 min at 4 °C. The supernatant fluid was then filtered using a 0.22 m filter (Millipore, USA) followed by centrifugation 100,000 × *g* for 120 min at 4 °C (Beckman 436C Optima X, Beckman Coulter, USA). For exosome purification, the pellets were washed thrice with 1× PBS and centrifuged at 100,000 × *g* for 120 min at 4 °C. Exosome pellets were collected, resuspended in 1 PBS, subpacked, and kept at −80 °C. Then, using a bicinchoninic acid (BCA) assay kit (Pierce, USA), protein quantification was done. TEM and NTA were used to examine the ultrastructure, concentration, and size distribution of exosomes.

We labeled the exosomes with PKH67 green fluorescent labeling kit (BestBio, BB-3986) to see if macrophages could take them up. The tagged exosomes were then injected into mice or cocultured with BEAS-2B cells. The lung tissues of mice were excised 6 hours after injection and examined with a laser-scanning confocal microscope (STELLARIS 5, Leica, Germany) to see if exosomes had reached the lung. BEAS-2B cells were stained with DAPI and viewed with a laser-scanning confocal microscope after the indicated duration of coculture.

### Transmission electron microscopy

Exosome pellets were loaded for 4 min at room temperature onto a formvar carbon-coated 200-mesh copper electron microscopy grid, then stained with phosphotungstic acid for 2 min at room temperature, dried, and visualized using a transmission electron microscope (TEM; JEM-1400, JEOL Ltd., Japan).

### Nanoparticle tracking analysis

We used NTA with the ZetaView PMX 110 (Particle Metrix, Meerbusch, Germany) and the accompanying software ZetaView 8.04.02 to measure exosome particle size and concentration. Briefly, isolated exosome samples were diluted with 1 PBS. At 11 different locations, NTA measurements were taken and evaluated. The ZetaView system was calibrated using polystyrene particles with a diameter of 110 nanometers.

### Endotoxin content assay test

The exosome samples were tested for endotoxin contamination using the Amplite^™^ Colorimetric Endotoxin Detection Kit (AAT Bioquest, Inc.). Endotoxin levels were found to be <0.1 EU/ml. When exosomes were added to cells, the endotoxin concentration was <0.001 EU/ml (Supplementary Fig 3), well below the minimum activation threshold of TLR4^51^.

### Proteomics of exosomes

The proteomic analysis was carried out using LC-MS/MS. A BCA kit was used accordingly as described by the manufacturer to determine protein content. The exosome solution was reduced for 30 min at 56 °C with 5 mM dithiothreitol, alkylated for 15 min at room temperature in the dark with 11 mM iodoacetamide, and then diluted for trypsin digestion. The peptides were then desalted using a Phenomenex Strata X C18 SPE column and vacuum centrifuged to dry them. The peptides were exposed to a nitrogen solubility index source before being subjected to tandem mass spectrometry (MS/MS) in a Q Exactive (Thermo Fisher Scientific, San Jose, CA, USA) linked online to an ultra-high-performance liquid chromatography system. The MaxQuant search engine (v.1.5.2.8) was used to process the MS/MS data.

### Bioinformatics analysis

The UniProt-GOA database (http://www.ebi.ac.uk/GOA/) was used to create the GO annotation proteome. Through InterProScan (a sequence analysis application), we annotate the functional descriptions of identified protein domains based on the protein sequence alignment approach, and the InterPro (http://www.ebi.ac.uk/interpro/) domain database was employed. Subcellular localization was predicted using Wolfpsort. The enrichment of differentially expressed proteins against all detected proteins was tested using a two-tailed Fisher’s exact test for each category. Against all identified proteins, the KEGG database was utilized to find the differentially expressed proteins’ enriched pathways.

### Enzyme-linked immunosorbent assay (ELISA)

The APN/CD13 and CRP concentrations in the exosomes isolated from plasma or media supernatant were determined using ELISA kits (EIAab, Wuhan, CN), Cytokine concentrations in plasma or BALF were determined using TNF-α and IL-6 ELISA kits (EIAab, Wuhan, CN), as described by the manufacturer. The concentrations of APN/CD13, CRP, TNF-α, and IL-6 were determined using a standard curve that was run for each ELISA.

### Cell culture

The Shanghai Cell Bank of the Chinese Academy of Sciences in China provided the mouse RAW264.7 macrophages and BEAS-2B cells. The cells were grown at 37 °C in a humidified atmosphere of 5% CO_2_ in DMEM (Life Technologies, Grand Island, NY, USA) supplemented with 10% fetal bovine serum (FBS, Life Technologies). Exosome-treated cells were grown in DMEM with 10% exosome-depleted FBS (Gibco, USA).

BMDMs were isolated as previously described^52^. The bone marrow obtained from mouse femurs and tibias was flushed with prechilled DMEM. After lysis, the cells were resuspended and cultured in DMEM with 10% FBS, 10 ng/mL M-CSF, and 50 g/mL penicillin/streptomycin at a concentration of 106 cells/mL in 60 mm dishes. On day 7, the BMDMs had been fully differentiated and were ready for use.

### Animal strains

The animal experimental protocol was approved by the Animal Welfare and Ethics Management Committee of the Southern Medical University and followed the Guide for the Care and Use of Laboratory Animals. C57BL/6 mice (male, 8–10 weeks old) were purchased from the Center of Experimental Animals of Guangdong Province.

### Cecal ligation and puncture model

CLP (cecal ligation and puncture) was performed as previously reported^53^. In a nutshell, the mice were sedated with an intraperitoneal injection of pentobarbital (60 mg/kg). The cecum was externalized, ligated with 4-0 silk sutures, and punctured once with a 22-gauge needle by one through-and-through puncture after a 1.5 cm abdominal incision. We then used 4:0 sutures to close the incision and internalize the cecum. The mice were observed for mortality every 6 h during the survival experiments. The mice were sacrificed 24 h after CLP surgery for some experiments, and their blood, BALF, and lungs were harvested.

### In vivo biodistribution of BMDM-derived exosomes

Two hundred microliters of exosomes derived from LPS-treated (1 μg/mL and 24 h) BMDMs were promptly injected into the CLP mice through the tail vein. To assess the tissue distribution of BMDM-derived exosomes in vivo, PKH67-labeled exosomes (100 μg/mouse) were introduced into the mice via tail vein, as described previously^54^. The exosomes were injected immediately after CLP operation, and the mice were sacrificed after 6 h of injection. The sectioned tissue of the lungs was stained with DAPI, and images were recorded through a fluorescence microscope (Leica DMi8).

### TEM for mitochondrial ultrastructure

The lung tissues or BEAS-2B cells were fixed with glutaraldehyde (2.5%) for 4 h before being rinsed in PBS. The tissues were dehydrated using alcohol gradients: 50, 70, 80, and 90% (15 min each), followed by 100% alcohol (15 min, twice), after being treated with 1% osmium tetroxide for 2 h. For the soaking process, a 3:1 epoxy propane soaking/embedding solution was used for 30 min, a 1:1 epoxy propane/embedding solution for 3 h, and a pure embedding solution was used overnight at ambient temperature. The samples were placed in an oven at 37 °C for 24 h and 60 °C for 48 h after being embedded in the capsule or embedding plate containing embedding agent respectively. The samples were then cut into ultrathin slices of 90 nm using an ultra-thin slicer Leica UC7 (Leica Microsystems Ltd., Germany). After 20 min of uranium dioxide acetate staining, the slices were stained with lead citrate for 10 min. Finally, the specimens were observed and photographed using TEM.

### Production of lentivirus and infection

OBiO (Shanghai OBiO Co., LTD) created lentiviral particles expressing shRNA against APN and the flanking control sequence. BMDMs and RAW264.7 cells were infected with lentiviral vectors, and GFP^+^ cells were selected using FACS for further experiments. APN expression was confirmed using qPCR and western blotting.

### Cell viability assays and apoptosis analysis

The Cell Counting Kit-8 (CCK8) assay (Dojindo, Japan) was used to determine cell viability. Following the protocols of an Annexin V-FITC/PI Cell Apoptosis Detection Kit (BD Biosciences, East Rutherford, NJ, USA), Annexin V-FITC binding solution and PI were added. FlowJo software (version 10.0.7; Tree Star, Inc., Ashland, OR, USA) was used to analyze the data.

### Chromatin immunoprecipitation assay

Immunoprecipitation of DNA-protein complexes was performed with BMDMs and RAW264.7, using the ChIP Kit (Millipore, Billerica, MA, USA) as guided by the manufacturer with antibodies c-Myc and normal mouse IgG (Millipore). Using particular primers (Supplementary Table 5), qPCR analysis was carried out for the precipitated DNA.

### Real-time quantitative PCR

RTRIzol (Gene Copoeia, MD, USA) was used to extract RNA from cells and tissues. Using specified primers and SYBR Green (TaKaRa Bio-tech), total RNA (1 μg) was reverse transcribed into cDNA and then subjected to real-time PCR to evaluate the genes. The particular primers (specified in Supplementary Table 6) were used in real-time PCR utilizing a Bio-Rad Real-Time PCR machine. The 2^−ΔΔct^ approach was used to calculate relative gene expression.

### Western blotting

Proteins were extracted using with RIPA lysis buffer containingbuffercontaining protease inhibitors (Sigma). The concentration of the extracted protein was quantified using with a BCA protein assay kit (Thermo). Equal amounts of total proteins (20 μg) were separated usingby 10% SDS-PAGE and transferred to a PVDF membrane (Millipore, Billerica, MA, USA). The membraneMembrane was blocked with 5% non-fat milk at room temperature for 2 h and incubated with the indicated antibodies. After incubation with the horseradish peroxidase-conjugated secondary antibody, the protein bands were visualized usingby enhanced chemiluminescence reagents (Merck Millipore). Primary antibodies, includingincluded CD63, CD81, c-Myc, CD13, TLR4, NF-κB p-p65, NF-κBp65, RelB, NF-κB1, NF-κB2, RIPK1, RIPK3, p-MLKL, and GAPDH, are listed in Supplementary Table 7. Images were captured using with a ChemiDoc imaging system (Bio-Rad).

### Co-immunoprecipitation (Co-IP)

Cells from 0.5 OD cultures were pelleted and resuspended within appropriate lysis buffer supplied with protease inhibitors and PMSF. To lower the background, 500 µL supernatant containing about 500–1000 µg protein was incubated with 5 µL protein A and 5 µL protein G for 1 h at 4 °C. Total protein lysates that removed unspecific binding protein were obtained after centrifugation. For protein binding, 1–5 µg corresponding antibodies (TLR4, antibodies’ information was as mentioned) and homologous IgG (eBiosciences, USA) were added to pre-cleaned protein lysates. Immediately afterward, samples were incubated at 4 °C overnight under gentle rotation. To precipitate target proteins, 5 µL protein A and 5 µL protein G were added to bind antigen–antibody complexes; the reaction was maintained at 4 °C for 3 h. After being gently washed three times with wash buffer, the unbound proteins were removed and the pellet (agarose–antibody–antigen complex) was resolved in 20–40 µL SDS-loading buffer for further western blotting with the indicated antibodies.

### Immunofluorescent staining

Cells or tissues were fixed for 30 min at room temperature in 4% paraformaldehyde, permeabilized for 5 min with 0.1% Triton X-100, and then blocked for 30 min at room temperature with 5% BSA. The cells or tissues were incubated overnight at 4 °C with the indicated primary antibodies: anti-RIP1, anti-RIP3, anti-NF-κB p65, anti-TLR4, and anti-CD13. The next day, the coverslips were subjected for incubation with fluorescent labeled secondary antibodies (1:100) for 1 h in a dark room. The incubated nuclei were stained with DAPI for 5 min. The samples were observed under a fluorescence microscope (STELLARIS 5, Leica, Germany).

### Immunohistochemical staining

TLR4, NF-κB p-p65, RIPK1, and RIPK3 proteins were detected using immunohistochemical assays on paraffin slices generated from in vivo investigations. As per the package instructions, the indirect streptavidin-peroxidase method was used. Two pathologists evaluated immunohistochemically stained tissue slices individually. Rabbit anti-TLR4, anti- NF-κB p-p65, anti-RIPK1, and anti-RIPK3 antibodies were employed.

### Hematoxylin and Eosin (H&E) staining and lung injury score

The lungs were fixed in 4% paraformaldehyde. After fixation, the lungs were embedded in paraffin, cut into 5 μm sections, and subjected to H&E staining according to routine histopathological methods. Histopathological changes were observed under a light microscope. The degree of lung injury (atelectasis, alveolar and interstitial inflammation, alveolar and interstitial hemorrhage, alveolar and interstitial edema, necrosis, and overdistension) was evaluated in six sections from the lower lobes using the following criteria: 0, no injury; 1, injury to 25% of the field; 2, injury to 50% of the field; 3, injury to 75% of the field; 4, diffuse injury. Lung injury was evaluated by independent pathologists who were blinded to the group allocation.

### TLR4-deficient mice

C57BL/6 (WT) and TLR4 knockout (C57BL/10ScNJGPt) mice were purchased from Jiangsu Jicui Yaokang Bio-Tech Co., Ltd. (license number: SCXK(Su)2018-0008). The mice were housed in experimental Animal Center (SPF grade), and the experiment was initiated after the mice acclimated to their environment. They were studied using protocols approved by the Ethics Committee of Guangdong Medical University.

### Proximity ligation assay

For PLA was performed according to the manufacture’ s protocol using the Duolink Detection Kit (DUO92102-1KT, Sigma-aldrich)^55,56^. BEAS-2B cells were plated in 24-well-plates, treated with exosomes as described above, then washed with PBS and fixed with 4% formaldehyde for 15 min and blocked with 5% BSA for 30 min, Then, TLR4, APN, RIP1, RIP3 antibodies were prepared with 5% BSA dissolved in 0.5% Triton-100 and incubated with cells at 4°Covernight. Subsequently, cells were washed and incubated with anti-rabbit (plus) and anti-mouse (minus) PLA probes for 1 h. Then, ligase solution was added for 30 min followed by an incubation with amplification- polymerase solution for 100 min. After washing steps with wash buffer and PBS, cells were incubated with a secondary antibody (goat anti-mouse Alexa Fluor 488) for 1 h at room temperature to immunolabel PLA target proteins. Probe incubation, ligation and amplification reaction were carried out according to the manufacturer instructions. Nuclei were stained using DAPI (1:1000, Sigma), and samples were mounted. PLA samples were analyzed by with a confocal microscope (objective×60, OLYMPUS). *p* value was calculated using Student’s *t* test. All experiments were performed at least three times with reproducible results.

### Luciferase reporter assay

To investigate the c-Myc impact on the activity of APN promoter, a 200 bp fragment containing the APN promoter’s three c-Myc binding sites was cloned into the pGL3-basic luciferase reporter vector, and c-Myc-binding site mutation vectors were created. These c-Myc plasmids and vectors were co-transfected into BMDMs and RAW264.7. The APN promoter’s luciferase activity was measured 48 h after transfection using the Dual-Luciferase Reporter Assay System (Promega Corporation, Madison, WI, USA).

### Ethics statement

Clinical data have been registered in the China Clinical Trials Registry. Collection of human blood was approved by the Medical Ethics Committee of the Shenzhen Hospital of Southern Medical University. All animal experiments conducted were compliant with Ethics Management Committee of the Southern Medical University.

### Statistical analysis

All data analyses were performed using the GraphPad Prism software version 8.0. The data are expressed as the mean ± SD from at least three independent experiments. Survival curves were constructed using the Kaplan–Meier method and analyzed using the log-rank test. Differences were considered statistically significant at *P* < 0.05. The results were analyzed using Student’s *t* test for two groups, one-way ANOVA analysis for multiple groups.

### Reporting summary

Further information on research design is available in the Nature Research Reporting Summary linked to this article.

## Supplementary information

Supplementary Information

Description of Additional Supplementary Files

Supplementary Data 1

Supplementary Data 2

Supplementary Data 3

Supplementary Data 4

Reporting Summary

## Data Availability

The source data for the graphs and charts in the main figures are provided in Supplementary Data 4. The original uncropped blot/gel images of the main figures are provided in Supplementary Fig. 4. The mass spectrometry proteomics data have been deposited to the ProteomeXchange Consortium via the PRIDE^57^ partner repository with the dataset identifier PXD032793 (Proteomics analysis) and PXD032815 (PRM). All other data are available from the corresponding author on reasonable request.
