# Supplementary material for: Macrophage-derived exosomal aminopeptidase N aggravates sepsis-induced acute lung injury by regulating necroptosis of lung epithelial cell
Source: Commun Biol. 2022 Jun 6;5:543. doi: 10.1038/s42003-022-03481-y (PMC9170685; doi:10.1038/s42003-022-03481-y)
Supplement: Supplementary file 1 — Supplementary Information [file 42003_2022_3481_MOESM1_ESM.pdf]

Supplementary Tables and Figures

Supplementary Tables

Supplementary Table 1. Baseline characteristics of healthy volunteers and sepsis patients for cohort 1:

	Healthy volunteers [a] n=5	Sepsis-induce ALI [a] n=5	*P* value
Age (years)	52.6 ± 5.8	54.0 ± 8.1	0.892
Gender, male, n (%)	3 (60%)	3 (60%)	
Body mass index (BMI)	20.0 ± 1.2	21.2 ± 1.0	0.460
Septic Shock (n)	/	3 (60%)	
Source of infection (n)			
Abdominal	/	3 (60%)	
Urogenital	/	2 (40%)	
Acute lung injury (n)	/	5(100%)	
SOFA score, median (IQR)	/	6.2 (4.5-8)	
APACHE II score, median (IQR)	/	18 (14-23)	

[a] original samples for proteomics analysis. The values are given as Mean ±SD or n (%). APACHE II: Acute Physiology and Chronic Health Evaluation score II; SOFA: Sequential Organ Failure Assessment.

Supplementary Table 2. Baseline characteristics of healthy volunteers and sepsis patients for cohort 2, which is independent patient pool from cohort 1.

	Healthy volunteers [b] n=15	Sepsis-induce ALI [b] n=13	*P* value
Age (years)	55.7 ± 2.9	61.9 ± 5.0	0.28
Gender, male, n (%)	8 (53%)	6 (46%)	
Body mass index (BMI)	22.0 ± 0.8	22.7 ± 0.8	0.56
Septic Shock (n)	/	5 (38%)	
Source of infection (n)			
Abdominal	/	8 (61%)	
Urogenital		3 (23%)	
Blood		1 (8%)	
Wound	/	1 (8%)	
Acute lung injury (n)	/	13 (100%)	
SOFA score, median (IQR)	/	6.5 (5-8)	
APACHE II score, median (IQR)	/	18.4 (15-23)	

[b] original samples for PRM. The values are given as Mean ±SD or n (%). APACHE II: Acute Physiology and Chronic Health Evaluation score II; SOFA: Sequential Organ Failure Assessment.

Supplementary Table 3. Baseline characteristics of healthy volunteers and sepsis patients for cohort 3, which is independent patient pool from cohort 1 and cohort 2.

	Healthy volunteers [c] n=36	Sepsis [c] n=16	Sepsis-induce ALI [c] n=61	*P* value
Age (years)	53.3 ± 2.3	53.2 ± 1.0	54.9 ± 1.6	0.79
Gender, male, n (%)	19 (53%)	11 (69%)	35 (57%)	
Body mass index (BMI)	22.7 ± 0.61	21.0 ± 0.66	21.4 ± 0.38	0.10
Septic Shock (n)	/	3 (19%)	41 (67%)	
Source of infection (n)				
Abdominal	/	10 (62%)	50 (82%)	
Urogenital		3 (19%)	6 (10%)	
Blood		2 (13%)	3 (5%)	
Wound	/	1 (6%)	2 (3%)	
Acute lung injury (n)	/	0	100 (100%)	
SOFA score, median (IQR)	/	6.1 (4-9)	8.1 (5-10)	**0.03**
APACHE II score, median (IQR)	/	17.1 (10-24)	20.5 (16-26)	0.08

[c] confirmatory samples for ELISA. The values are given as Mean ±SD or n (%). APACHE II: Acute Physiology and Chronic Health Evaluation score II; SOFA: Sequential Organ Failure Assessment.

Supplementary Table 4. Demographic and clinical characteristics of survivors and non-survivors

	Survivors n=22	Non-survivors n=16	*P* value
Age (years)	55.4 ± 3.1	60.0 ± 2.9	0.31
Gender, male, n (%)	10 (45%)	10 (63%)	
Body mass index (BMI)	21.9 ± 0.62	20.4 ± 0.64	0.11
Septic Shock (n)	11 (50%)	10 (63%)	0.52
Source of infection (n)			
Abdominal	16 (73%)	12 (75%)	
Urogenital	3 (13%)	2 (13%)	
Blood	2 (9%)	1 (6%)	
Wound	1 (5%)	1 (6%)	
Acute lung injury (n)	100 (100%)	100 (100%)	
SOFA score, median (IQR)	7 (5-9)	11(9-13)	**< 0.001**
APACHE II score, median (IQR)	18 (12-25)	22 (19-26)	**0.04**

The values are given as Mean ±SD or n (%). APACHE II: Acute Physiology and Chronic Health Evaluation score II; SOFA: Sequential Organ Failure Assessment.

Supplementary Table 5. The primers used in this study

Primers name		Sequence (5’-3’)
IL-6	Forward	CCACCGGGAACGAAAGAGAA
	Reverse	TCTCCTGGGGGTATTGTGGA
TNF-α	Forward	AGAACTCACTGGGGCCTACA
	Reverse	GCTCCGTGTCTCAAGGAAGT
APN/CD13	Forward	ATTGGGCAAGGTCTGGTCTG
	Reverse	GAGAAGGAGAACGAGCCACC
CHIP-APN- sitA	Forward	TCCTCAAAAGATGCCCTG
	Reverse	CCAACTGTTCTGCCTCTT
CHIP-APN- sitB	Forward	GAGACAGGGAGCCCCACC
	Reverse	GGAGTTTTAGTAGAGACA
CHIP-APN- sitC	Forward	TGAGAAGCGCGTGTTCCC
	Reverse	AGCCTCGCCCTGAGTGTC
c-Myc	Forward	CTGGATTTTTTTCGGGGTAGT
	Reverse	ACGCACAAGAGTTCCGTA

Supplementary Table 6. The sequences used in this study

Gene		Sequence (5'-3')
Si-c-Myc1	Forward	GCUUCACCAACAGGAACUATT
	Reverse	UAGUUCCUGUUGGUGAAGCTT
Si-c-Myc2	Forward	GUGCAGCCGUAUUUCUACUTT
	Reverse	AGUAGAAAUACGGCUGCACTT
Negative control	Forward	UUCUCCGAACGUGUCACGUTT
	Reverse	ACGUGACACGUUCGGAGAATT
shAPN1	Forward	CcggGCAGGACTACTGGCTGATATTCAAGAGAATTAACC TCGCTGTACTGGTTTTTTg
	Reverse	aattcaaaaaaGCAGGACTACTGGCTGATATCTCTTGAAATTA ACCTCGCTGTACTGG
shAPN2	Forward	CcggCCAGTACAGCGAGGTTAATTTCAAGAGAATAGCGT TGCAGTAGACGGTTTTTTg
	Reverse	aattcaaaaaaCCAGTACAGCGAGGTTAATTCTCTTGAAATAG CGTTGCAGTAGACGG
shAPN3	Forward	CcggCCGTCTACTGCAACGCTATTTCAAGAGAATAGCGTT GCAGTAGACGGTTTTTTg
	Reverse	aattcaaaaaaCCGTCTACTGCAACGCTATTCTCTTGAAATAG CGTTGCAGTAGACGG
shNC	Forward	CcggCCTAAGGTTAAGTCGCCCTCGCTCGAGCGAGGGCG ACTTAACCTTAGGTTTTTTg
	Reverse	aattcaaaaaaCCTAAGGTTAAGTCGCCCTCGCTCGAGCGAGG GCGACTTAACCTTAGG

Supplementary Table 7. A list of antibodies used for Western blot, IHC staining, IF, Co-IP, CHIP.

Name of antibody	Cat.No	Company	Mol weight	Dilution (WB/ IHC/ CHIP)
CD63	ab59479	Abcam	52 kDa	1:1000 (WB)
CD81	ab79559	Abcam	26 kDa	1:1000 (WB)
c-Myc	ab32072	Abcam	57 kDa	1:1000 (WB); 1:50 (CHIP)
F4/80	28463-1-AP	proteintech		1:100 (IF)
CD13/APN	ab108310	Abcam	150 kDa	1:1000 (WB)；1:100 (IF)
TLR4	ab13556	Abcam	96 kDa	1:1000(WB); 1:50 (IF)<br>1:100 (IP); 1:50 (IHC)
TLR4 (76B357.1)	sc-52962	Santa Cruz Biotechnology	96 kDa	1:1000(WB); 1:50 (IF)
NF-κB p65	55764T	CST	65 kDa	1:1000 (WB)；1:100 (IF)
P-NF-κB p65	55764T	CST	65 kDa	1:1000 (WB)；1:100 (IF)<br>1:200 (IHC)
RelB	55764T	CST	70 kDa	1:1000 (WB)
NF-κB1 p105/p50	55764T	CST	50, 120 kDa	1:1000 (WB)
NF-κB2 p100/p52	55764T	CST	120, 52 kDa	1:1000 (WB)
p-IκB-α (B-9)	sc-8404	Santa Cruz Biotechnology	36 kDa	1:1000 (WB)
IκB-α (H-4)	sc-1643	Santa Cruz Biotechnology	36 kDa	1:1000 (WB)
RIPK1	ab268944	Abcam	75 kDa	1:1000 (WB); 1:100 (IF)
RIP1	3493	CST	75 kDa	1:1000 (WB); 1:100 (IP)<br>1:100 (IF)
RIPK3	A5431	Abclonal	57 kDa	1:1000 (WB); 1:200 (IHC)<br>1:100 (IF)

Phospho-RIP (Ser166)	44590	CST	75 kDa	1:1000 (WB)
RIP3	17563-1-AP	proteintech	55 kDa	1:100 (IP)； 1:1000 (WB)<br>1:100 (IF)
MLKL	21066-1-AP	proteintech	55 kDa	1:1000 (WB)
P-MLKL	74921S	CST	55 kDa	1:1000 (WB)
Cleaved Caspase-3	9664	CST	17 kDa	1:1000 (WB)
Caspase-3	9662	CST	37 kDa	1:1000 (WB)
Caspase-8	4790	CST	57 kDa	1:1000 (WB)
Cleaved Caspase-8	8592	CST	43 kDa	1:1000 (WB)
GAPDH	5174	CST	37 kDa	1:1000 (WB)
Tubulin	ab184966	Abcam	55 kDa	1:1000 (WB)

Supplementary Figures and Figure Legends

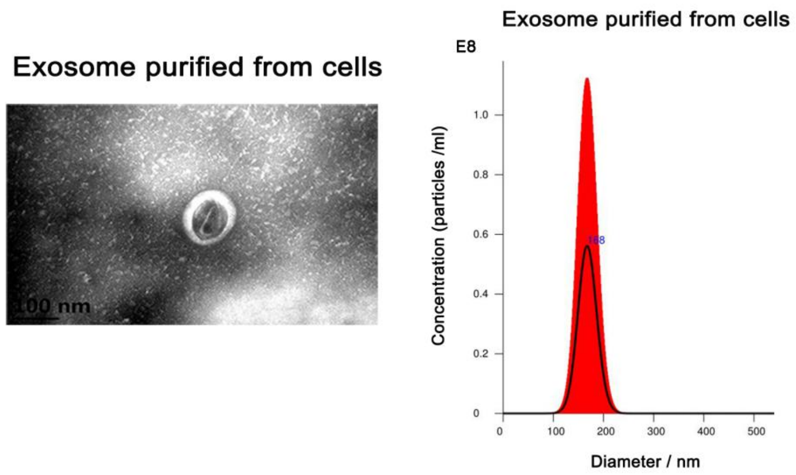

Supplementary Figure 1 Supplementary figures were related to Figure 3. Exosomes were purified from these supernatants and identified via TEM and NTA.

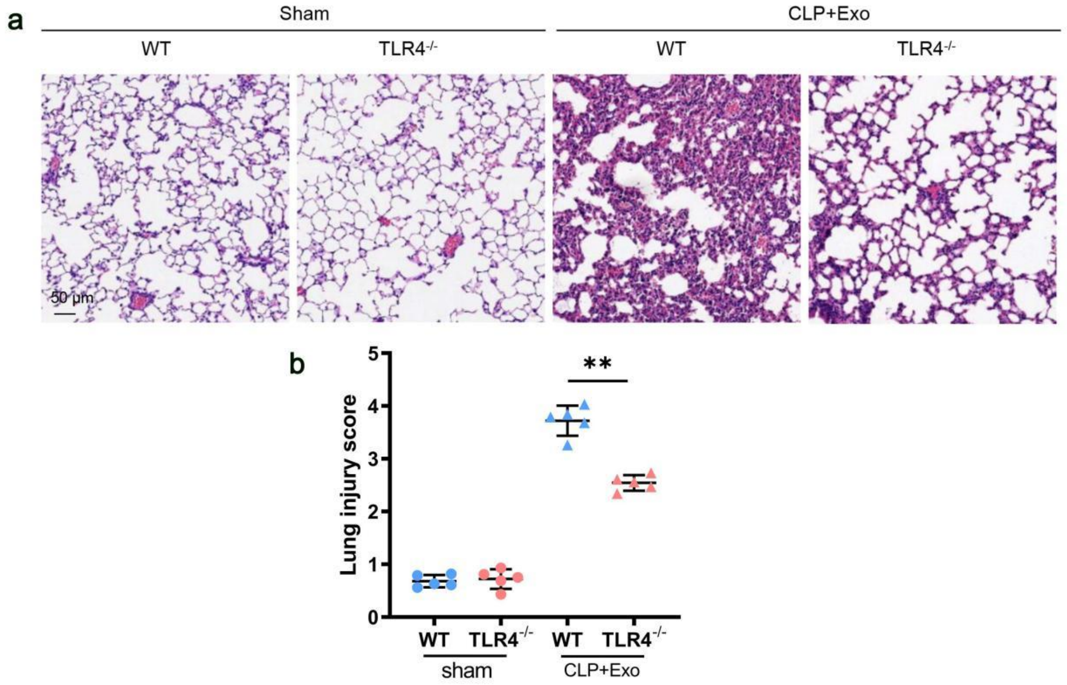

Supplementary Figure 2 Supplementary figures were related to Figure 6. The WT and TLR4 knockout（KO）mice were subjected to sham or CLP and injected with LPS-exo (APN in the purified exosomes). a Representative H&E staining images of mice lung tissues. Scale bar, 50 μm. b Lung injury score was evaluated due to the pathology. All results are based on three measurements. The information is presented as the mean ± SD, *$P < 0.05$, **$P < 0.01$.

The exosome samples were tested for endotoxin contamination. Endotoxin levels were found to be less than 0.01 EU/ml in both PBS-exo and LPS-exo.

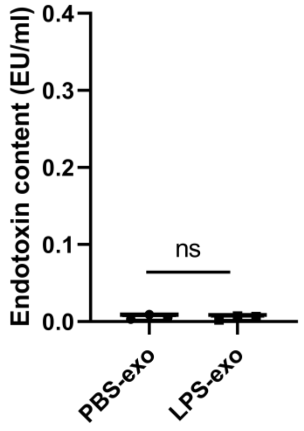

Supplementary Figure 3 Endotoxin levels in the exosomal preparations. LPS-exo and PBS-exo represent exosomes isolated from LPS-treated BMDMs than PBS-treated BMDMs, respectively. Data are expressed as the mean ± SEM.

Supplementary Figure 4 The original uncropped blot/gel images of the main figures.

Fig 1c

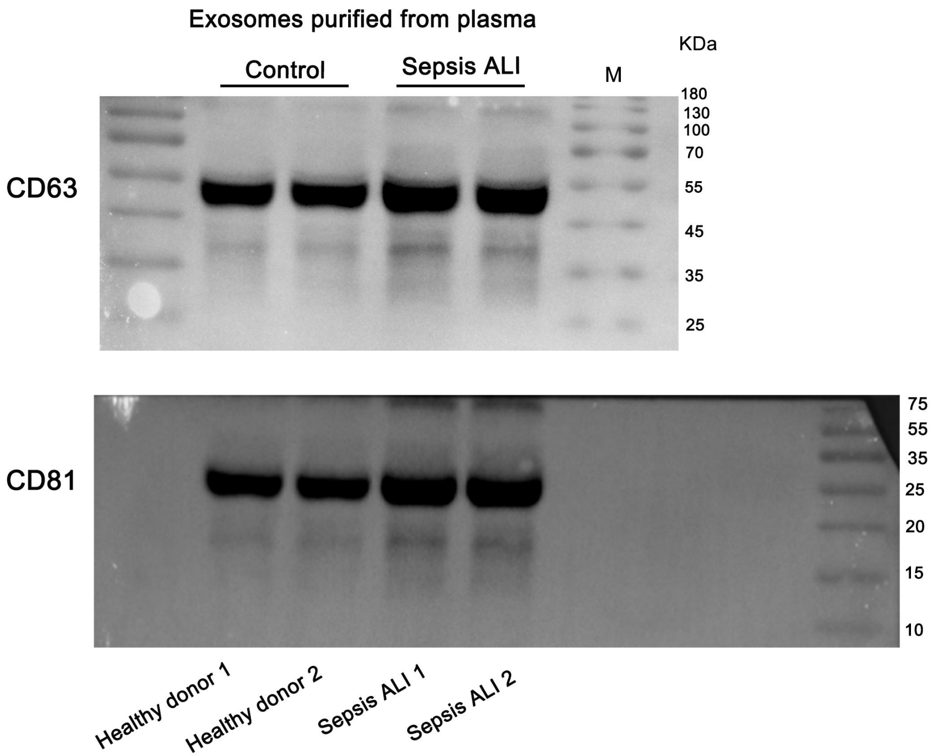

Fig 3c

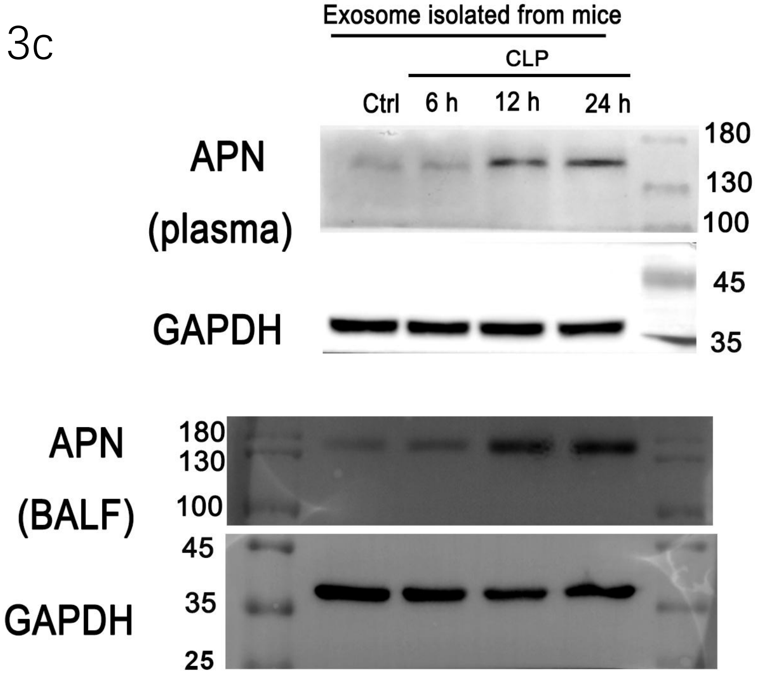
Exosome isolated from mice
CLP
Ctrl
6 h
12 h
24 h
APN
(plasma)
180
130
100
45
35
GAPDH
APN
(BALF)
180
130
100
45
35
25
GAPDH

Fig 3g

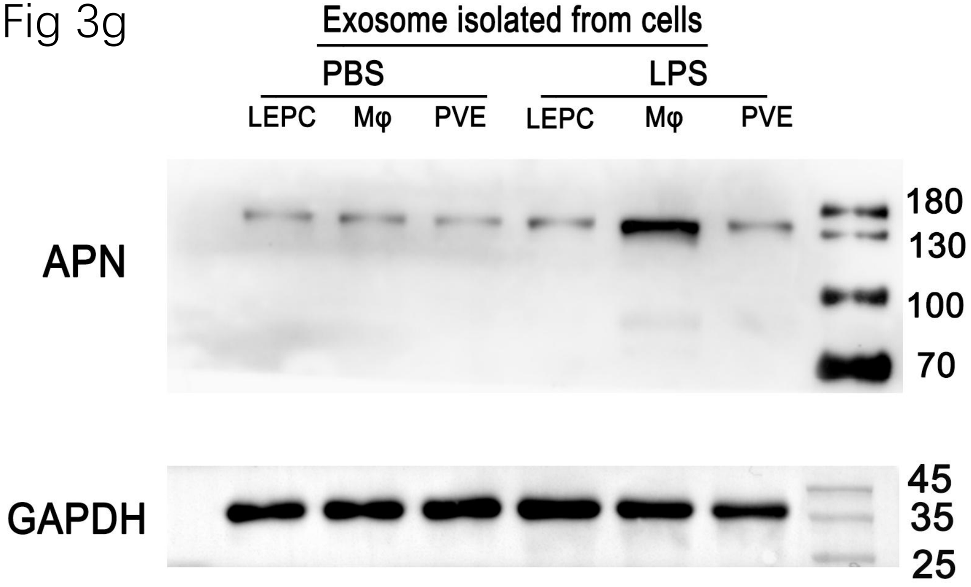
Exosome isolated from cells
PBS
LPS
LEPC
Mφ
PVE
LEPC
Mφ
PVE
APN
180
130
100
70
GAPDH
45
35
25

Fig 3j

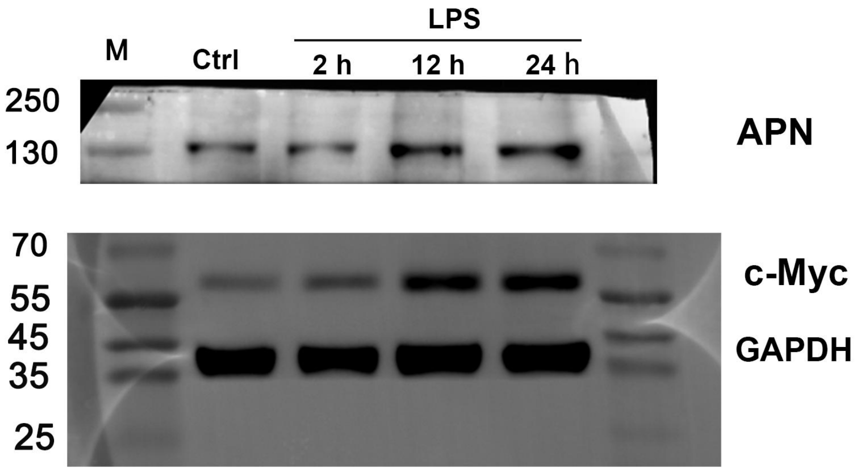

Fig 3k

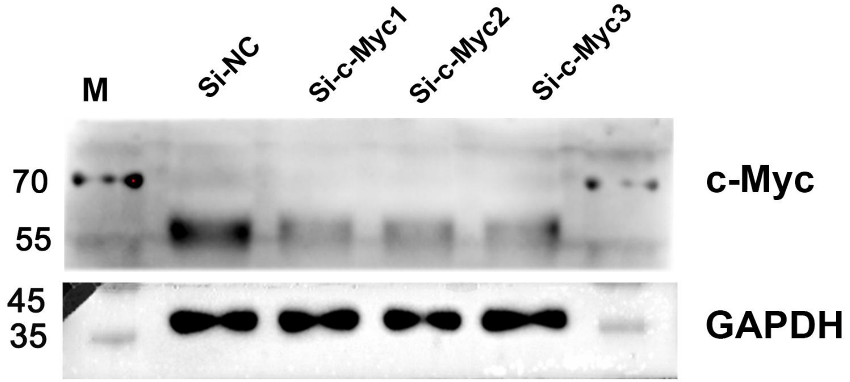

Fig 3l

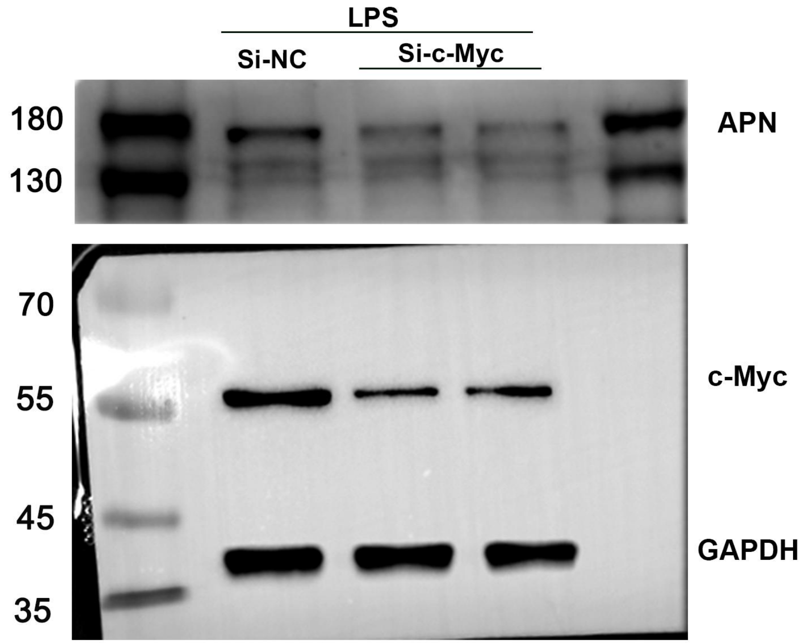

Fig 3o

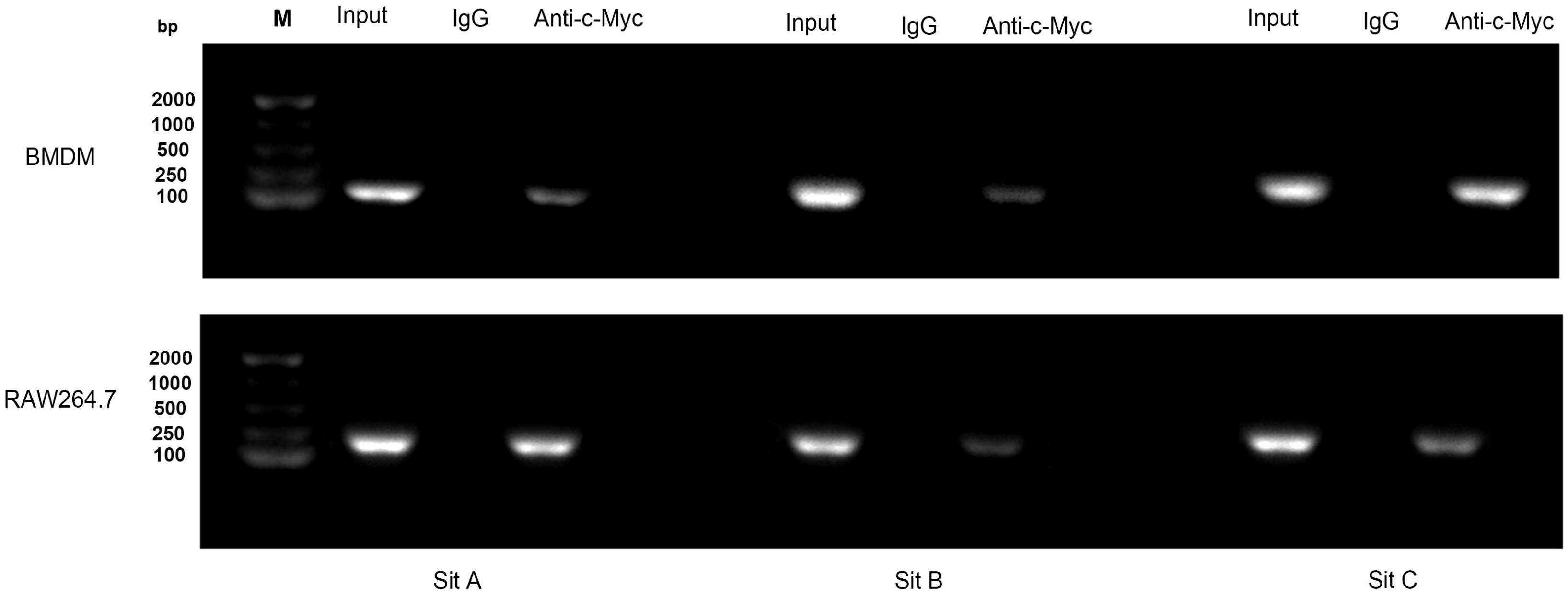
bp
M
Input
IgG
Anti-c-Myc
Input
IgG
Anti-c-Myc
Input
IgG
Anti-c-Myc
BMDM
2000
1000
500
250
100
RAW264.7
2000
1000
500
250
100
Sit A
Sit B
Sit C

Fig 4d

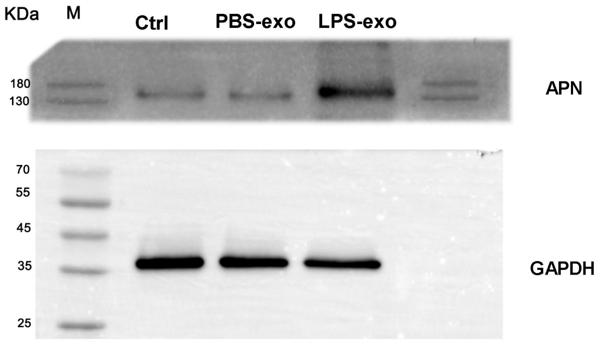
KDa
M
Ctrl
PBS-exo
LPS-exo
180
130
APN
70
55
45
35
25
GAPDH

Fig 4h

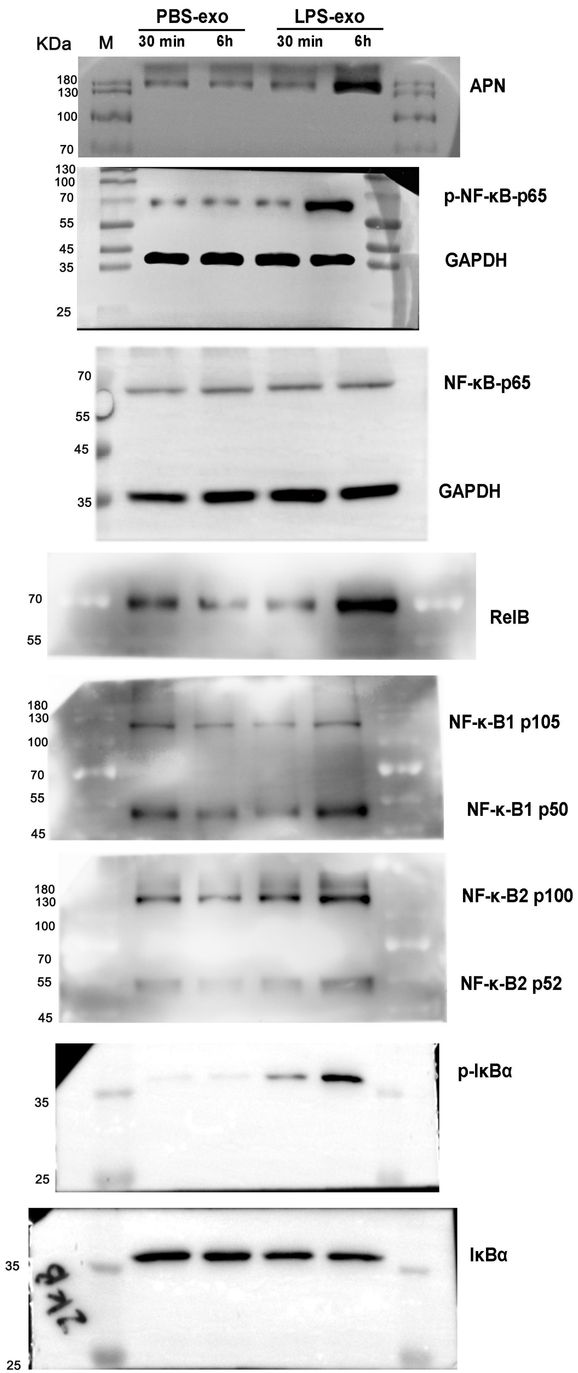
KDa
M
PBS-exo
LPS-exo
30 min
6h
30 min
6h
180
130
100
70
APN
130
100
70
55
45
35
25
p-NF-κB-p65
GAPDH
70
55
45
35
NF-κB-p65
GAPDH
70
55
RelB
180
130
100
70
55
45
NF-κ-B1 p105
NF-κ-B1 p50
180
130
100
70
55
45
NF-κ-B2 p100
NF-κ-B2 p52
35
25
p-IκBα
35
25
IκBα

Fig 4l

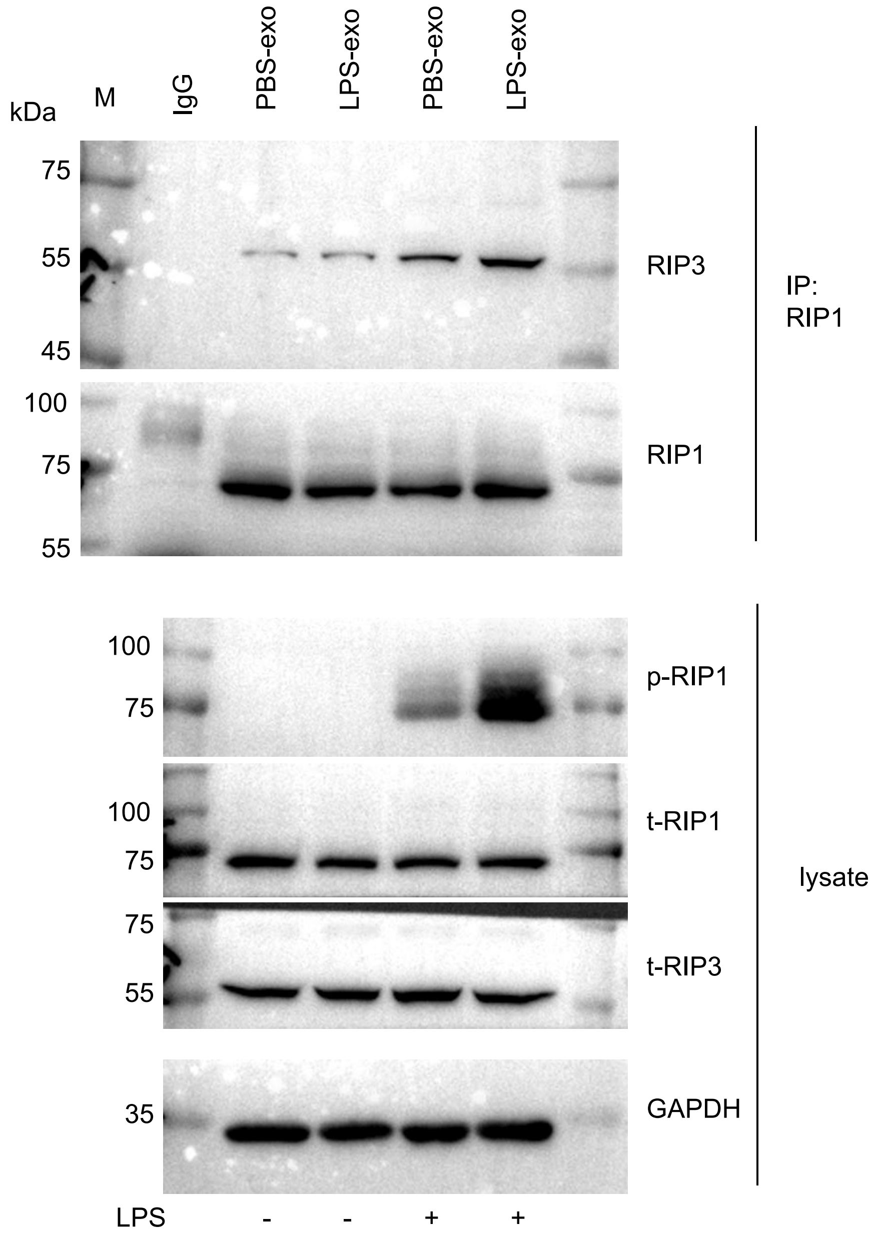
kDa
M
IgG
PBS-exo
LPS-exo
PBS-exo
LPS-exo
75
55
45
RIP3
IP:
RIP1
100
75
55
RIP1
100
75
p-RIP1
100
75
t-RIP1
lysate
75
55
t-RIP3
35
GAPDH
LPS
-
-
+
+

Fig 4n

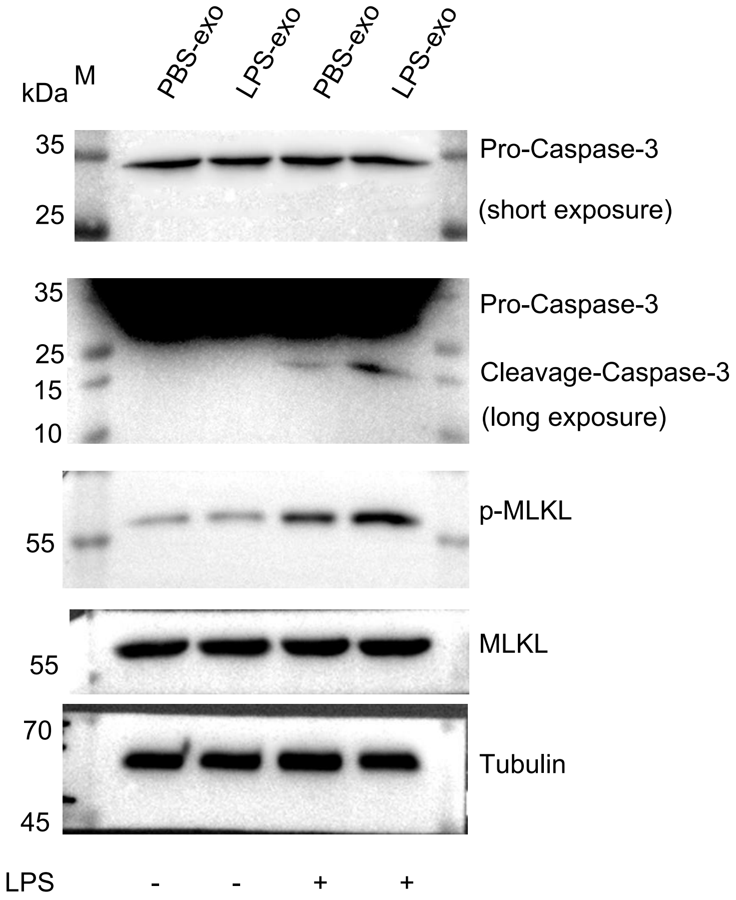
kDa
M
PBS-exo
LPS-exo
PBS-exo
LPS-exo
35
25
Pro-Caspase-3
(short exposure)
35
25
15
10
Pro-Caspase-3
Cleavage-Caspase-3
(long exposure)
55
p-MLKL
55
MLKL
70
45
Tubulin
LPS
-
-
+
+

Fig 5a

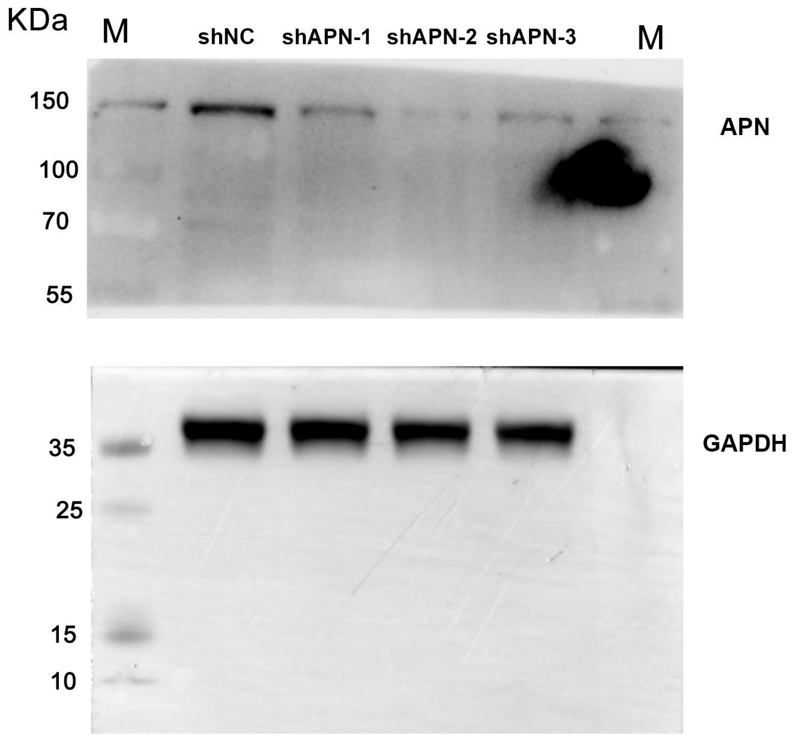

KDa
M
shNC
shAPN-1
shAPN-2
shAPN-3
M
150
100
70
55
APN
35
25
15
10
GAPDH

Fig 5d

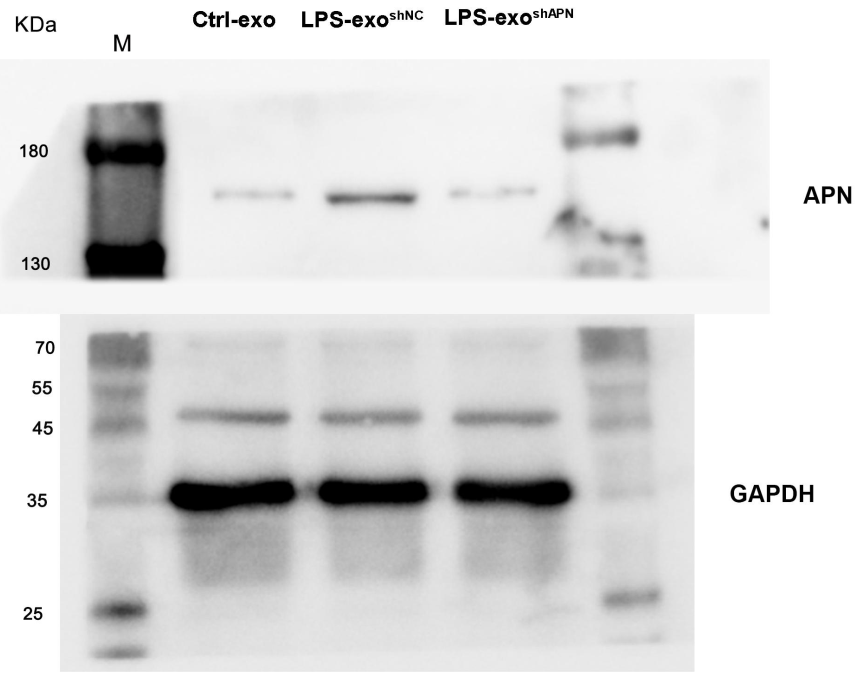

KDa
M
Ctrl-exo
LPS-exo^shNC
LPS-exo^shAPN
180
130
APN
70
55
45
35
25
GAPDH

Fig 5h

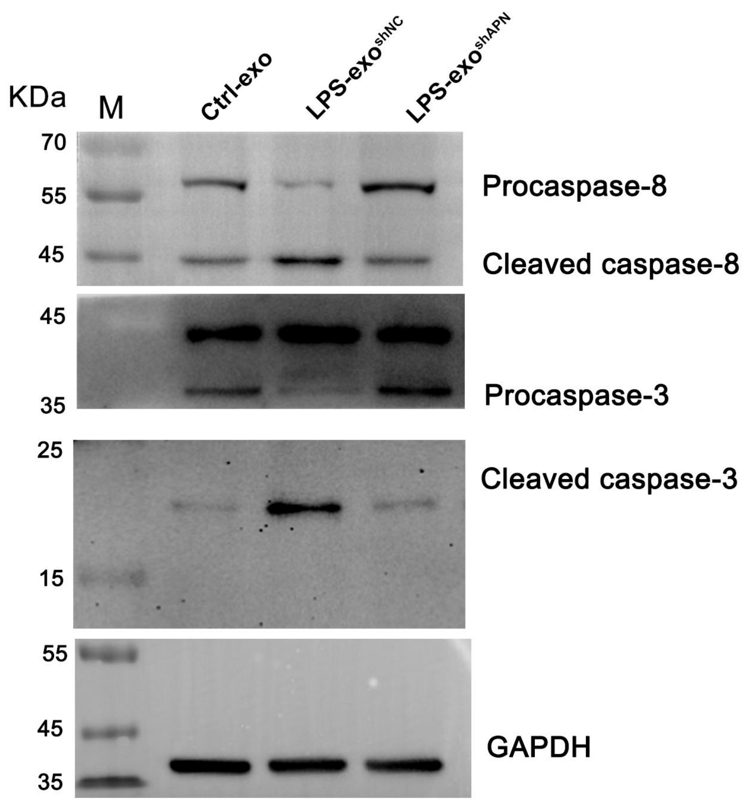
KDa
M
Ctrl-exo
LPS-exo shNC
LPS-exo shAPN
70
55
45
Procaspase-8
Cleaved caspase-8
45
35
Procaspase-3
25
15
Cleaved caspase-3
55
45
35
GAPDH

Fig 5l

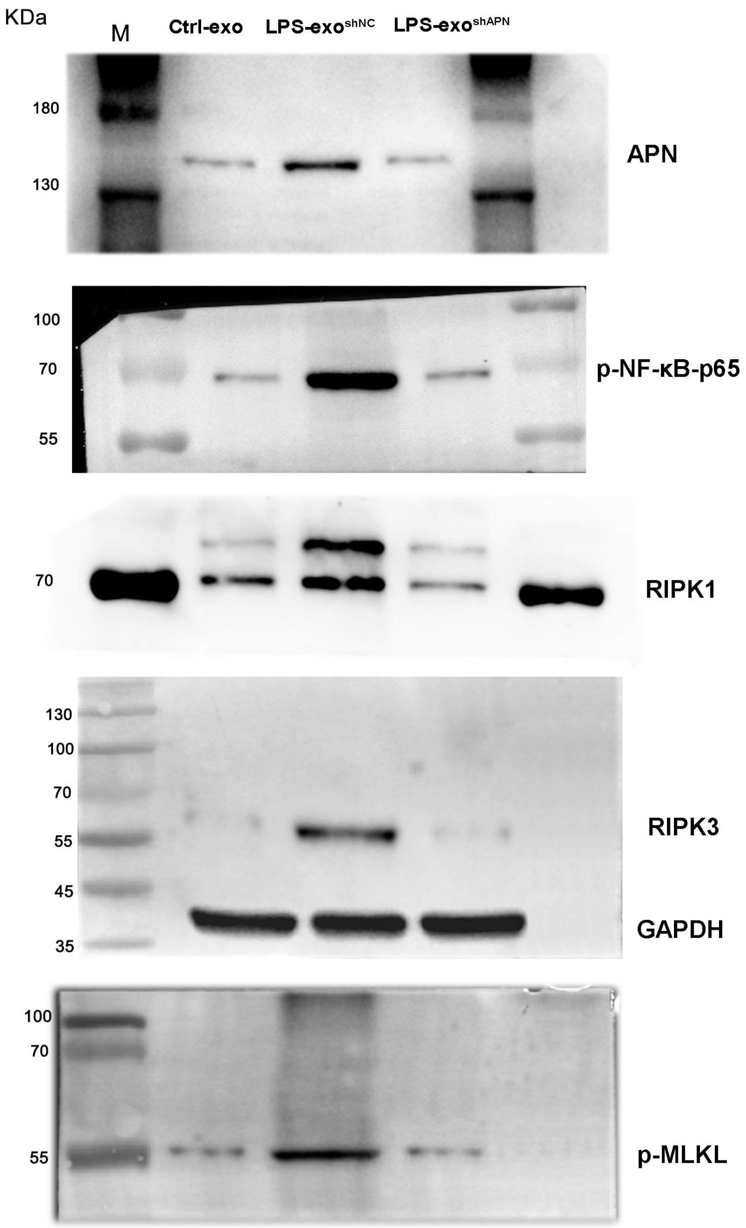
KDa
M
Ctrl-exo
LPS-exo shNC
LPS-exo shAPN
180
130
APN
100
70
55
p-NF-κB-p65
70
RIPK1
130
100
70
55
45
35
RIPK3
GAPDH
100
70
55
p-MLKL

Fig 5n

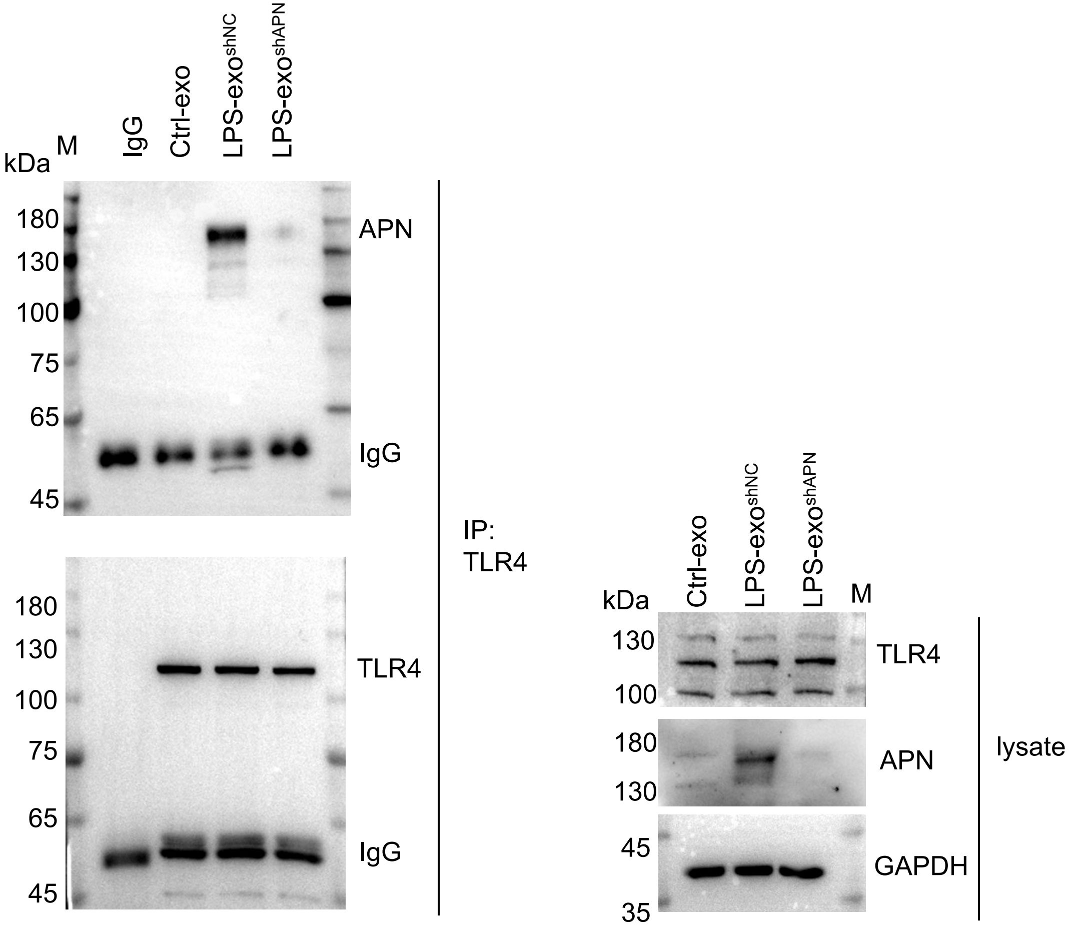
kDa
M
IgG
Ctrl-exo
LPS-exo^shNC
LPS-exo^shAPN
180
130
100
75
65
45
APN
IgG
180
130
100
75
65
45
TLR4
IgG
IP:
TLR4
kDa
Ctrl-exo
LPS-exo^shNC
LPS-exo^shAPN
M
130
100
TLR4
180
130
APN
45
35
GAPDH
lysate

Fig 6k

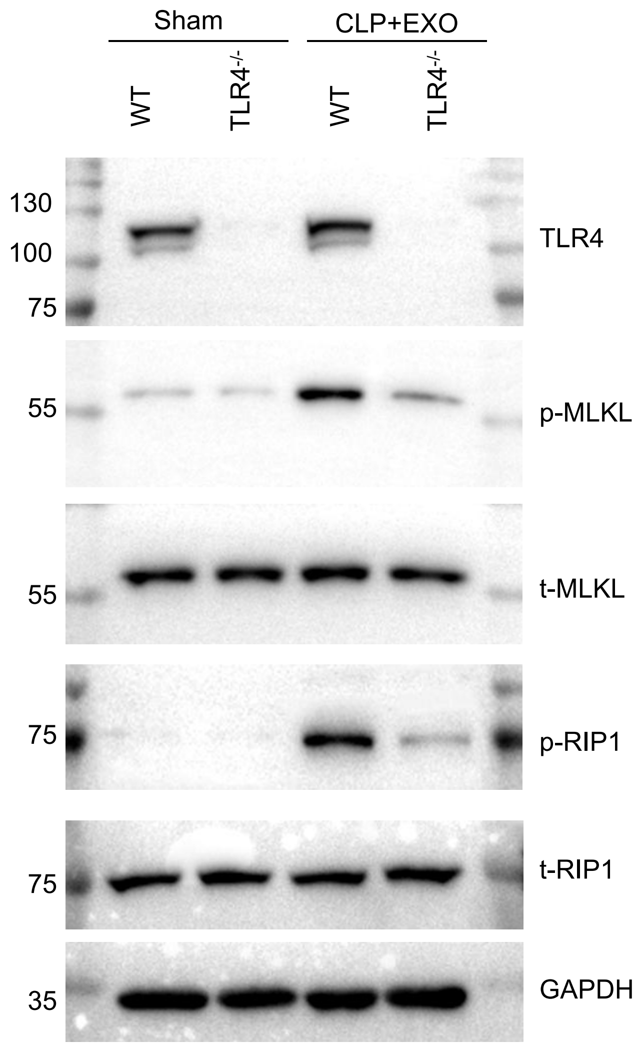
Sham
CLP+EXO
WT
TLR4-/-
WT
TLR4-/-
130
100
75
TLR4
55
p-MLKL
55
t-MLKL
75
p-RIP1
75
t-RIP1
35
GAPDH
