# Supplementary material for: Macrophage-derived exosomal aminopeptidase N aggravates sepsis-induced acute lung injury by regulating necroptosis of lung epithelial cell
Source: Commun Biol. 2022 Jun 6;5:543. doi: 10.1038/s42003-022-03481-y (PMC9170685; doi:10.1038/s42003-022-03481-y)
Supplement: Supplementary file 2 — Description of Additional Supplementary Files [file 42003_2022_3481_MOESM2_ESM.pdf]

Description of Additional Supplementary Files

File name: Supplementary Data 1
Description: MS_identified_information

File name: Supplementary Data 2
Description: Fold_1.5 differentially_expressed_protein

File name: Supplementary Data 3
Description: The data for PRM

File name: Supplementary Data 4
Description: The source data for the graphs and charts in the main figures
